# Comparative evaluation of specimen type and processing conditions for studying oyster microbiomes

**DOI:** 10.3389/fmicb.2024.1504487

**Published:** 2025-01-08

**Authors:** Esam Almuhaideb, Nur A. Hasan, Christopher Grim, Shah Manzur Rashed, Salina Parveen

**Affiliations:** ^1^Department of Agriculture, Food and Resource Sciences, University of Maryland Eastern Shore, Princess Anne, MD, United States; ^2^Center for Bioinformatics and Computational Biology, University of Maryland, College Park, MD, United States; ^3^EzBiome Inc, Gaithersburg, MD, United States; ^4^Center for Food Safety and Applied Nutrition, U.S. Food and Drug Administration, College Park, MD, United States; ^5^Cosmos ID, Germantown, MD, United States

**Keywords:** oyster microbiome, shotgun metagenomics, *Crassostrea virginica*, mollusk, *Vibrio* spp.

## Abstract

Metagenomic sequencing is increasingly being employed to understand the assemblage and dynamics of the oyster microbiome. Specimen collection and processing steps can impact the resultant microbiome composition and introduce bias. To investigate this systematically, a total of 54 farmed oysters were collected from Chesapeake Bay between May and September 2019. Six different specimen types and processing methods were evaluated for microbial community composition using shotgun metagenomics, namely fresh oyster homogenate (FOH), oyster homogenate after simulated temperature abuse (AOH), Luria broth-enriched oyster homogenate (EOH), dissected stomach homogenate (DSH), hemolymph (HLM), and stomach-gut content (SGC). In general, DSH, EOH, and FOH yielded the highest DNA concentration, while EOH had the highest microbial reads, followed by DSH, HLM, and FOH. HLM produced the highest bacterial species alpha diversity, followed by AOH, EOH, and SGC. Although alpha diversities did not differ significantly, beta-diversity measurements showed significant dissimilarity among methods (*p* < 0.05) indicating that the specimen types and processing steps do play an important role in representing the composition of the bacterial community. Bacterial species that had the highest log mean abundance included *Cyanobium* sp. PCC 7001 in FOH, *Vibrio vulnificus* in AOH, EOH, and DSH, and lastly *Synechococcus* sp. CB0205 in the DSH, HML, and SGC samples. EOH displayed higher bacterial hits, distinct microbial composition, and higher values of bacterial, phages, and antimicrobial resistance gene reads. Therefore, if studying the overall oyster microbial community, prioritizing optimum specimen collection and processing methods that align with the overall goal of the study is recommended.

## Introduction

Foodborne illnesses caused by pathogenic *Vibrio*, specifically *Vibrio parahaemolyticus* and *V. vulnificus*, are commonly associated with the consumption of raw and undercooked shellfish, particularly oysters. In spite of the comprehensive *Vibrio* Control Plans under the National Shellfish Sanitation Program (NSSP), which encompass risk assessment, and mechanisms like hazard analysis and critical control points to manage *Vibrio* infection risk in shellfish production, incidents of illnesses related to oysters persist (U.S. Food and Drug Administration, 2024). Understanding the presence of pathogenic *Vibrio* species and their relative abundance within the oyster microbiome could provide foundational knowledge for future research and risk management efforts.

Routinely, foodborne pathogens in oysters are detected by culturing the species of interest using selective and differential growth media, followed by identification through techniques such as molecular or biochemical assays. Once the species is identified, its genetic information can be characterized using various methods such as sequencing of specific gene targets (PCR) or whole genomes (Brown et al., 2019). While this approach can provide valuable information on the identity and genetic makeup of the pathogen, it does not assess its relative abundance within the microbial community in oysters. Metagenomic sequencing is a culture-independent technique that interrogates the whole microbial community and overcomes this limitation. Next Generation Sequencing (NGS) techniques including 16s rDNA and Shotgun sequencing can be used to directly sequence the nucleic acid (DNA/RNA) of microbial communities in their natural habitats (Quince et al., 2017). These techniques have been shown to be highly sensitive in determining the composition and diversity of the complex microbial communities in oysters, with short turn-around times (Quince et al., 2017).

Metagenomic studies of the oyster microbiome are increasingly being employed to understand the assemblage of microbial communities and their interactions with pathogenic species associated with foodborne illnesses. Different types of oyster species have been studied including the Pacific oyster (*Crassostrea gigas*), Cortez oyster (*Crassostrea corteziensis*), Kumamoto Oyster (*Crassostrea sikamea*) (Trabal Fernández et al., 2014), and eastern oyster (*Crassostrea virginica*) (Ossai et al., 2017). Trabal Fernández et al. (2014) reported that Proteobacteria, Bacteroidetes, Actinobacteria, and Firmicutes in addition to 13 other phyla and 243 genera comprised the microbiomes of *Crassostrea gigas*, *C. corteziensis*, and *C. sikamea*. Furthermore, Proteobacteria was the dominant phylum of the oysters’ microbiota followed by Bacteroidetes (Trabal Fernández et al., 2014). Another study conducted in Hackberry Bay and Lake Caillou, Louisiana, revealed detailed analyses of *Crassostrea virginica* stomach and gut microbiome compositions (King et al., 2012). It showed variation in richness and composition between stomach and gut microbiome (King et al., 2012). This variation between tissues was consistent regardless of the collection sites and replicates (King et al., 2012). Microbial communities in the oyster stomach and gut were dominated by Mollicutes and Chloroflexi phyla, respectively. Stomach and gut microbiomes in other oyster replicate samples were dominated by Planctomycetes phylum and *Shewanella*, respectively (King et al., 2012), indicating oyster to oyster variation.

Recently, our research group reported that temperature influenced the diversity of oyster (*Crassostrea virginica*) microbiome in the Chesapeake Bay (Ossai et al., 2017). During the month of May, the microbial community of the oysters from Chester and Manokin, Maryland was dominated by *Pelagibacteraceae* family with 13.3 and 28.5% relative abundances, respectively (Ossai et al., 2017). While in June, the relative abundance of *Enterobacteriaceae* was 25.5 and 57.5%; *Synechococcus* was 35.7 and 16.2%, and *Pelagibacteraceae* was only 0.2 and 0.1%, respectively (Ossai et al., 2017).

Most of the studies previously conducted using NGS focused on the 16s rDNA amplicon approach (Chen et al., 2016; Chen et al., 2013; Chen et al., 2017; Ossai et al., 2017; Romero et al., 2002; Trabal Fernández et al., 2014). Although it excels in providing insights into the taxonomic diversity of bacterial communities, sequencing the 16s rDNA gene is specific for bacteria and primarily provides taxonomic resolution at the phylum to genus levels. Shotgun sequencing, on the other hand, is an unbiased technique that can provide whole genome content resolution of the dominant members of the microbial community, allowing taxonomic resolution at the species and strain level, yielding significant insights into the phylogenetic composition, species divergence, genotype profile and oyster’s microbiome response to the biotic and abiotic factors (Wooley et al., 2010). However, shotgun sequencing of samples such as seafood can be very challenging because these samples typically contain a large proportion of host cells. As a result, DNA sequencing typically returns with a massive excess of host DNA reads in addition to reads coming from microbiome DNA. Thus, ensuring that shotgun metagenomic studies employ effective sample preparation techniques is vital for maximizing the return of microbiome DNA reads.

Different oyster processing techniques have been reported for microbial analysis. Common techniques include homogenization of whole oyster, targeting a broader overview of oyster’s microbial community (Horodesky et al., 2020; Kaysner et al., 2004; Ossai et al., 2017; Parveen et al., 2020); tissue-specific dissection, providing localized insights (Drake et al., 2007; King et al., 2012; Li et al., 2017; Trabal Fernández et al., 2014); and microbial enrichment, improving microbial biomass representation (Kaysner et al., 2004; Parveen et al., 2020; Pathirana et al., 2019). Specimen type and processing condition can have a significant impact on sequencing outcome and thus can impact the relevance of microbiome profiling to the research question. It has been reported that the microbial community varies from oyster to oyster and between oyster specimens (Ossai et al., 2017; Trabal Fernández et al., 2014; Walton et al., 2013). Previous studies of oyster microbiomes using 16s rDNA amplicon sequencing indicated that the host site analyzed such as stomach, gut, hemolymph, or other internal components can be a strong determinant of bacterial community composition (King et al., 2012; Li et al., 2017; Lokmer et al., 2016; Pimentel et al., 2021). For instance, it has been reported that the gut communities of the eastern oysters were different from other tissues such as the stomach, mantle, gill, and inner shell (King et al., 2012; Pimentel et al., 2021). Another study reported that α-diversity was significantly different between pacific oyster tissues (gut, gills, mantle, and hemolymph), with the gut being significantly lower in diversity while the hemolymph had the highest species richness (Lokmer et al., 2016). This variation may be influenced by the physiological processes specific to each host site (Paillard et al., 2022). Moreover, temperature stress can alter the microbiome of oysters (Green et al., 2019; Lokmer and Mathias Wegner, 2015). A previous study indicated that oysters acclimated to two different temperatures, one group at 8°C (cold acclimation) and another group at 22°C (warm acclimation), exhibited significant alterations in the microbial dynamics and composition of their hemolymph communities when subjected to temperature stress in the opposite direction (Lokmer and Mathias Wegner, 2015). Therefore, investigating specimen type and processing steps is critical for understanding and characterizing the biases that influence microbiome diversity.

The aim of this study was to investigate how six different processing approaches, combining four specimen types, and two processing conditions influence the outcomes of shotgun metagenomic sequencing of the eastern oyster microbiome from the Chesapeake Bay.

## Materials and methods

### Study location and sampling

Eastern oysters (*Crassostrea virginica*) were collected from Tangier Sound (37°58′10.0″N 75°53′01.6″W) in the Chesapeake Bay once a month throughout May, June, August, and September 2019 (Supplementary Figure S1). For each sampling event, 12–15 oysters were harvested, placed into a plastic collection bag, and put into a cooler with ice using a sheet of bubble wrap to ensure no direct contact between the ice and oysters (Table 1). A Smart Button Data Logger was used to confirm that the temperature during transportation was lower than 10°C (U.S. Food and Drug Administration, 2024).

**Table 1:** Number and type of oyster samples.

Sample type	No. of oysters per event	Sampling events	Total
Fresh-oyster homogenate (FOH)^*^	3	4	12
Temperature abused-oyster homogenate (AOH)^*^	3	3	9
Enriched-oyster homogenate (EOH)^*^	3	4	12
Dissected stomach homogenate (DSH)^*^	3	3	9
Stomach gut contents (SGC)^*^	3^**^	3	9
Oyster-hemolymph (HLM)^*^	3^**^	4	12

* FOH and DSH represent the oyster and oyster gut microbiome; HLM and SGC are tissue-free specimens representing the hemolymph and gut microbiomes of oyster; AOH and EOH were used to increase microbial biomass in their natural and enriched environment.

** Hemolymph and stomach gut contents were collected from the same oyster.

### Processing of oyster samples

Oysters were cleaned at the laboratory using a scrub brush and tap water before they were shucked with sterile knives. Oysters were divided into six specimens and treatment conditions, with three replicates of each group. (1) Fresh oyster homogenate (**FOH**) buffered in 1:1 (w/v) Phosphate Buffer Saline (PBS) was obtained using a stomacher bag. This method has been successfully employed in previous studies to analyze the microbiomes of various oyster species (Chauhan et al., 2014; Diner et al., 2023; Horodesky et al., 2020; Madigan et al., 2014; Pathirana et al., 2019). (2) Oysters were placed at 25°C for 24 h, then homogenized and buffered in 1:1 (w/v) PBS using a stomacher bag to simulate temperature-abused oyster homogenate (**AOH**). Temperature stress is known to alter microbiome composition, as observed previously in microbial dynamics under different temperature conditions (Lokmer and Mathias Wegner, 2015). (3) Oysters were homogenized using a sterile blender for 1 min, and enriched (**EOH**) in Luria Broth (LB) at a ratio of 1:1 (w/v) and blended for another 1 min, then incubated at 37°C up to 10 h. Enrichment enhances the growth of microbial biomass and thus the detection sensitivity of microbial taxa that may be present in low abundance but are of significant interest, such as pathogenic bacteria. A short enrichment period was used to improve microbial biomass representation while minimizing biases associated with longer incubation times. (4) Oysters were dissected, and the oyster stomachs were homogenized (**DSH**) and buffered in 1:1 (w/v) PBS using a stomacher bag. Studies have shown that different oyster tissues harbor unique microbial communities, with recent research focusing on a variety of oyster organs, including digestive organs (Green and Barnes, 2010; King et al., 2012; Li et al., 2017; Lokmer et al., 2016; Pathirana et al., 2019; Pierce and Ward, 2019). (5) The hemolymph (**HLM**) and (6) stomach gut content (**SGC**) of three oysters were collected using a sterile syringe and 18-gauge needle. Oyster shells were drilled aseptically with a 5/8″ diameter hole saw directly above the adductor muscle with an approximate depth of 1 mm to be able to extract the hemolymph without disrupting the tissue. After hemolymph extraction, the oyster was shucked, and the gut contents of the stomach were collected. In addition to the widespread studies of oyster hemolymph and gut to understand the microbiome of the digestive and circulatory systems (King et al., 2012; Li et al., 2017; Lokmer and Mathias Wegner, 2015; Pathirana et al., 2019; Pierce and Ward, 2018, 2019), our research utilized hemolymph (HLM) and stomach gut contents (SGC) as tissue-free samples, aiming to minimize contamination from oyster tissues. Aliquots of 1–1.5 mL of each treatment replicate were collected and stored at −80°C for DNA extraction.

### Total DNA extraction, library preparation, and sequencing

Saponin treatment (0.025% Saponin) was performed before metagenomic DNA extraction to deplete oyster DNA. Initially, 1 mL of each sample was centrifuged at 2,000×*g* for 30 s to separate the supernatant from the pellet containing host cells. The supernatant was carefully transferred to a new tube, and subsequent centrifugation at 13,000×g for 10 min was performed. The pellet was resuspended in 400 μL 1× PBS and centrifuged at 13,000×g for 10 min. The pellet was resuspended again in 190 μL 1× PBS and 5 μL of 1% saponin was added to adjust the final concentration at 0.025% v/v Saponin and incubated for 10 min at room temperature. Following saponin treatment incubation, the samples were vortexed, and then 20 μL of 10× Turbo DNase buffer were added. The suspension was gently vortexed, then 2 μL Turbo DNase enzyme was added, well mixed, and incubated at 37°C for 1 h. Twenty microliter resuspended DNase inactivation reagent was then added, well mixed, and incubated for 5 min at room temperature. The sample was centrifuged at low speed to pellet the DNase inactivation buffer, and the clear supernatant was transferred to the Qiagen Power Soil Pro beating tube. DNeasy PowerSoil Kit (Qiagen, Germantown, MD, USA) was used to extract DNA following the manufacturer’s recommendation (Hermans et al., 2018; King et al., 2012; Pathak et al., 2023). DNA concentration was measured using a NanoDrop spectrophotometer (Thermo Fisher Scientific, USA). Preparation of sequencing libraries was performed using Nextera XT DNA Library Preparation Kit (Illumina Inc., San Diego, CA, USA) following the manufacturer’s protocol. Sequencing was performed using the Illumina HiSeq X (Illumina Inc., San Diego, CA USA), achieving a sequencing depth ranging from 20 to 40 million reads, which has been reported to be sufficient for comprehensive coverage of the microbial community (Bloomfield et al., 2023; Pereira-Marques et al., 2019). All oyster samples were processed and sequenced individually to preserve the unique microbial profiles of each replicate.

### Microbial detection and antibiotic resistance/virulence factors prediction

FASTQ files generated were analyzed by the CosmosID-HUB Microbiome bioinformatics software package (CosmosID, 2022). The system uses a data-mining k-mer algorithm that quickly separates millions of short DNA sequence reads into the specific genomes that produced them. The system has two phases, a pre-computation phase that uses curated databases of reference genomes, virulence markers, and antibiotic resistance markers to create a phylogeny tree of microbes and sets of k-mer fingerprints associated with different branches and leaves of the tree (CosmosID, 2022). The second part is a per-sample computation phase, where the system searches hundreds of millions of short sequence reads against the fingerprint sets to accurately detect and classify microorganisms in the sample (CosmosID, 2022). To prevent false positive identifications, the system applies a filtering threshold based on internal statistical scores that are determined by analyzing a wide range of metagenomes (CosmosID, 2022). This approach was also used to accurately detect genetic markers for virulence and antibiotic resistance.

### Statistical analysis

The alpha diversity of microbiome richness and evenness for oysters within each sample type was assessed using the Shannon index. To determine if there were significant differences in alpha diversities between the sample types, a non-parametric Wilcoxon Rank Sum test was used for pairwise comparisons, and a significance level of *p* < 0.05 was used (CosmosID, 2022). To evaluate the similarities of the microbial community between sample types (beta diversity), the Bray-Curtis index was utilized. Statistical significance in beta diversities was determined using permutational multivariate analysis of variance (PERMANOVA) (CosmosID, 2022). Furthermore, the abundance distribution and any significant differences in individual taxa with respect to sample type were identified using the Wilcoxon Rank Sum test (CosmosID, 2022). Linear Discriminant Analysis (LDA) effect size (LEfSe) was applied to identify biomarkers showing statistically significant and biologically consistent differences among the oyster sample types (LDA score > 2; *p* < 0.05) (CosmosID, 2022). Log abundance transformation was performed to visualize less dominant community members more easily.

## Results

### DNA yield of the oyster samples

**Figure 1:**
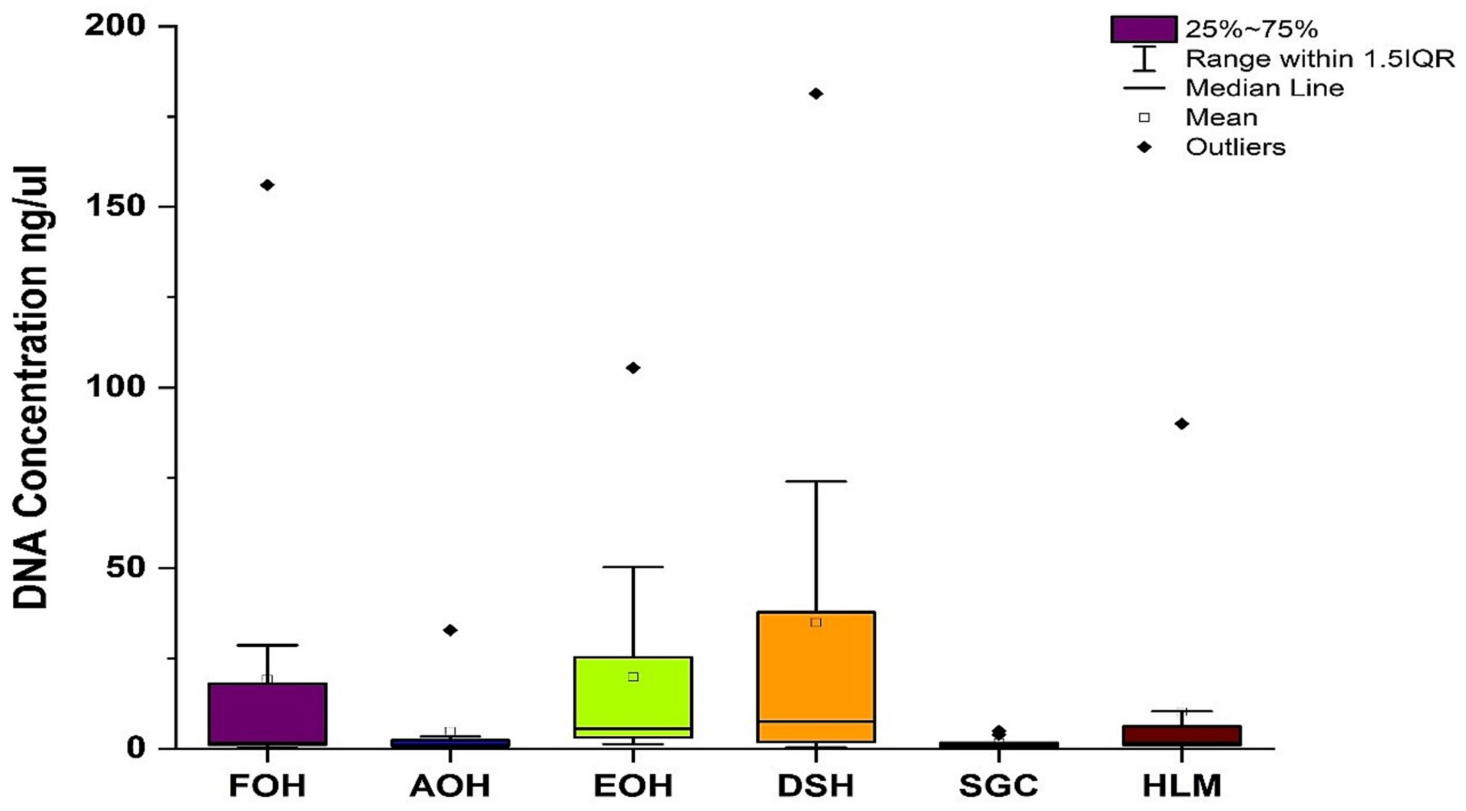
Concentration of DNA isolated from each sample type. FOH, Fresh-oyster homogenate; AOH, Temperature abused-oyster homogenate; EOH, Enriched-oyster homogenate; DSH, Dissected stomach homogenate; SGC, Stomach gut contents; HLM, Oyster-hemolymph.

The yields of DNA concentration varied between the oyster samples and ranged from 0.4 to 156 ng/μL in the FOH samples, 0.3–33 ng/μL in the AOH samples, 1.3–106 ng/μL in the EOH samples, 0.4–181 ng/μL in the DSH samples, 0.2–5 ng/μL in the SGC samples, and from undetectable-90 ng/μL in the HLM samples (Figure 1). The means of the DNA concentration were greatly higher than the medians indicating the variation between replicates (Table 2). Furthermore, there was a variation between the means of the sample types ranging from 2 to 35 ng/μL, while the medians of the samples were less variant with values ranging from 1 to 7.6 ng/μL (Table 2). Both DSH and EOH samples yield the highest means and medians of the DNA concentration.

**Table 2:** The average and median of the DNA yield and raw/hits reads per sample type.

Method outcomes	Avg.	FOH	AOH	EOH	DSH	SGC	HLM
	Med.						
DNA yield (ng/μL)		19	5	20	35	2	10
		1.65	1.20	5.55	7.60	1.0	1.70
Raw reads		43,518,126	**51,968,945**	39,353,883	37,590,617	31,244,906	41,418,696
		37,514,288	**55,497,278**	36,783,959	37,898,120	38,847,116	37,539,776
Bacterial hits		1,570,486	602,189	**3,347,573**	2,484,066	996,209	2,138,756
		869,000	410,000	**2,038,000**	556,000	91,390	426,500
Phages hits		35,704	323,039	**459,612**	36,980	110,096	130,405
		19,750	44,409	**230,500**	10,975	110,000	111,500
Viruses hits		12,848	**35,685**	29,043	8,583	10,946	21,130
		6,191	9,808	6,546	8,248	**10,824**	7,210
Protists hits		23,304	708	**39,537**	12,633	2,875	5,818
		**14,031**	373	3,940	11,540	2,483	3,674
Fungi hits		12,244	2,586	**13,039**	8,640	2,422	5,578
		8,471	2,466	4,027	**9,564**	1,704	3,573
ARGs hits		149	30	**1,123**	112	112	204
		76	20	**145**	25	28	41
VFGs hits		600	639	**715**	573	417	653
		**394**	36	242	229	78	346
All hits		1,655,477	964,917	**3,890,715**	2,551,697	1,123,122	2,302,647
		1,046,001	632,945	**2,314,679**	597,982	284,438	588,468
Microbial hits		1,654,728	964,248	**3,888,877**	2,551,012	1,122,593	2,301,790
		1,045,406	632,837	**2,314,361**	597,719	284,348	588,183

ARGs, antibiotic resistant genes; VFGs, virulence factor genes; FOH, Fresh-oyster homogenate; AOH, Temperature abused-oyster homogenate; EOH, Enriched-oyster homogenate; DSH, Dissected stomach homogenate; SGC, Stomach gut contents; HLM, Oyster-hemolymph.Bolded values indicate the highest values compared to other sample types.

### Composition and diversity of the oyster microbiome

#### Shotgun metagenomic sequencing of oyster samples

Shotgun metagenomic sequencing of all oyster samples resulted in over 2.5 billion raw reads; however, only just over 121 million (M) of the raw reads (5%) were assigned to bacterial species of the oyster microbiome in all sample types, despite saponin treatment. Among the sample types, the EOH displayed the highest percentage of bacterial hits (8.5%) for the sequencing reads. The other sample types ranked from highest to lowest were as follows: DSH (6.6%), HLM (5.2%), FOH (3.6%), SGC (3.2%), and AOH (1.2%).

#### Abundance and ubiquity of bacterial species among sample types

**Figure 2:**
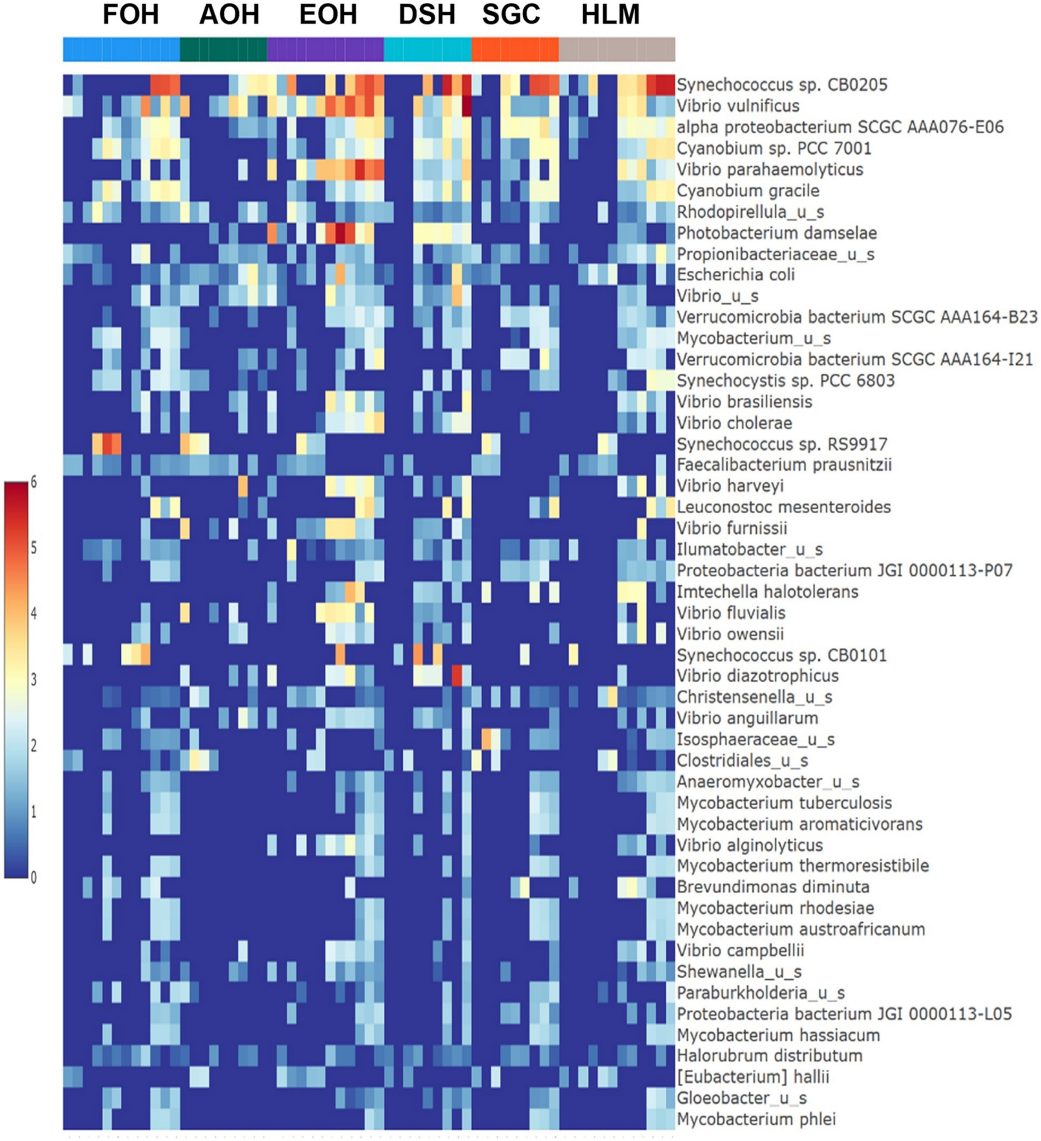
Log mean abundance of bacterial taxa within all sample types. FOH, Fresh-oyster homogenate; AOH, Temperature abused-oyster homogenate; EOH, Enriched-oyster homogenate; DSH, Dissected stomach homogenate; SGC, Stomach gut contents; HLM, Oyster-hemolymph.

**Figure 3:**
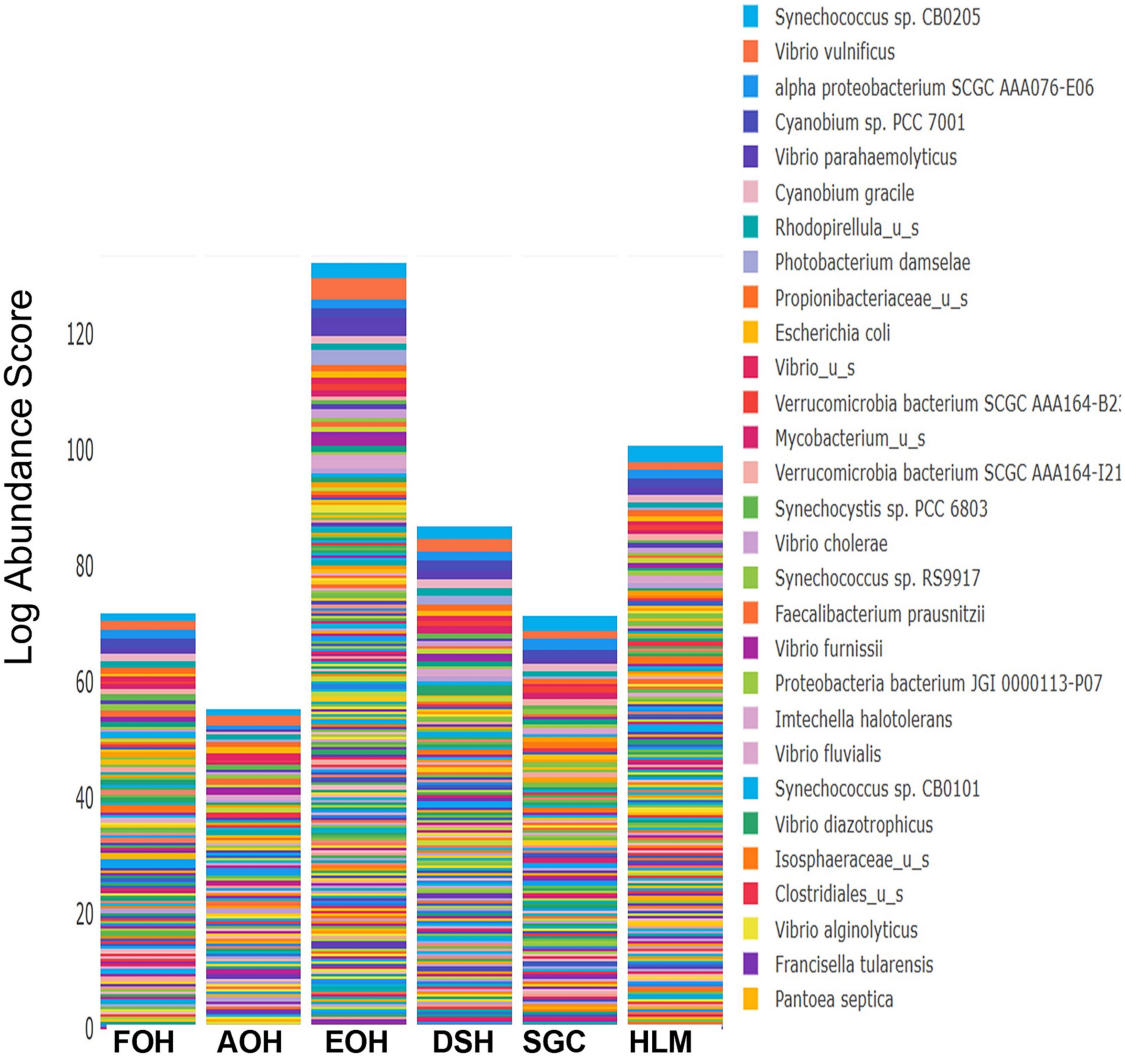
Relative log abundance score of bacterial taxa in relation to sample type. FOH, Fresh-oyster homogenate; AOH, Temperature abused-oyster homogenate; EOH, Enriched-oyster homogenate; DSH, Dissected stomach homogenate; SGC, Stomach gut contents; HLM, Oyster-hemolymph.

**Figure 4:**
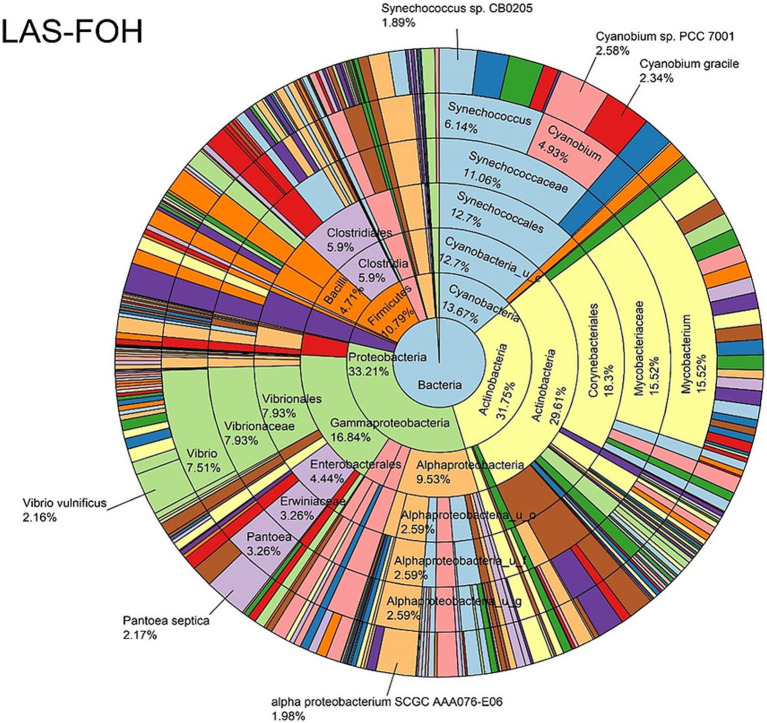
Log abundance distribution of bacterial taxa in the FOH samples. LAS, log abundance score; FOH, Fresh-oyster homogenate.

**Figure 5:**
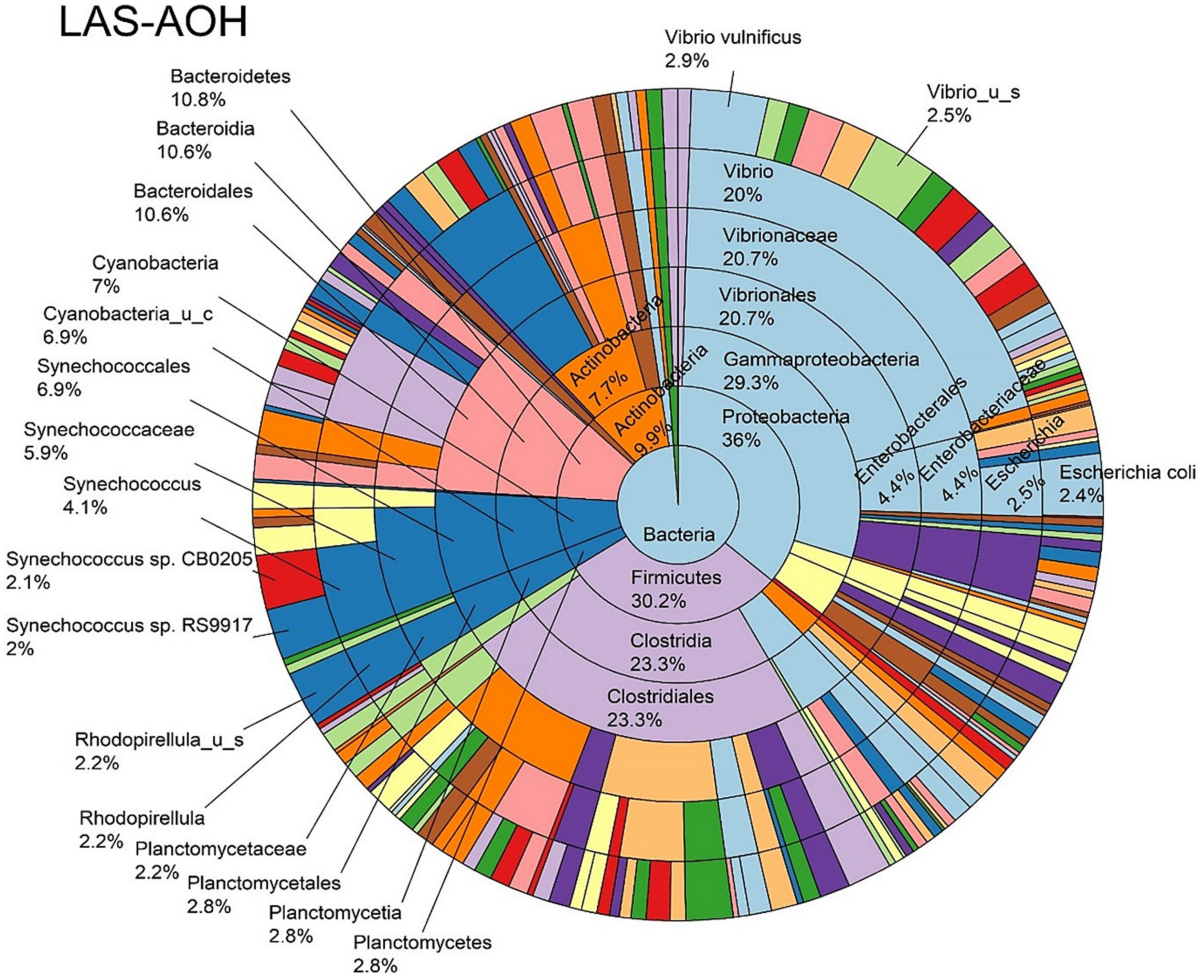
Log abundance distribution of bacterial taxa in the AOH samples. LAS, log abundance score; AOH, Temperature abused-oyster homogenate.

**Figure 6:**
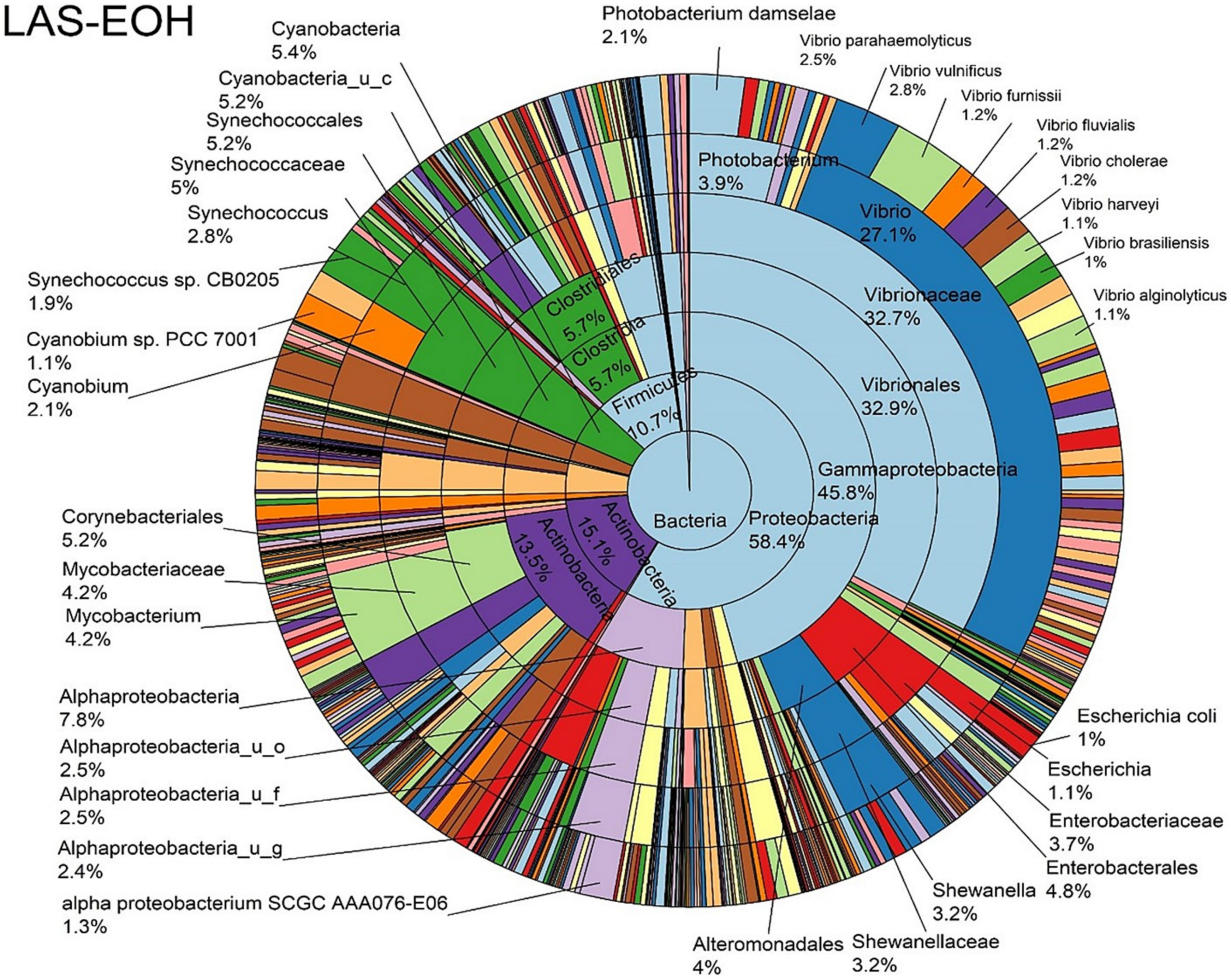
Log abundance distribution of bacterial taxa in the EOH samples. LAS, log abundance score; EOH, Enriched-oyster homogenate.

**Figure 7:**
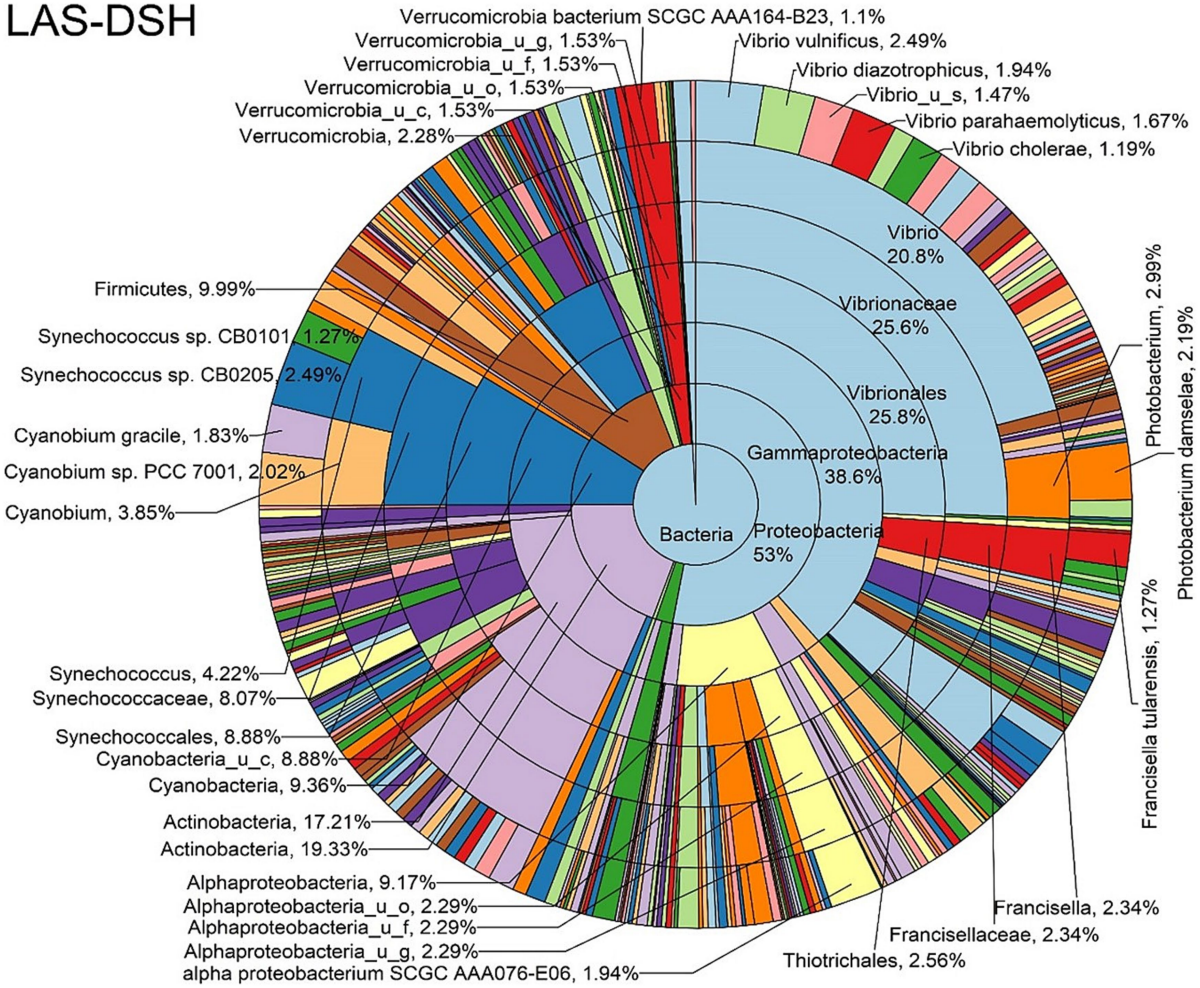
Log abundance distribution of bacterial taxa in the DSH samples. LAS, log abundance score; DSH, Dissected stomach homogenate.

**Figure 8:**
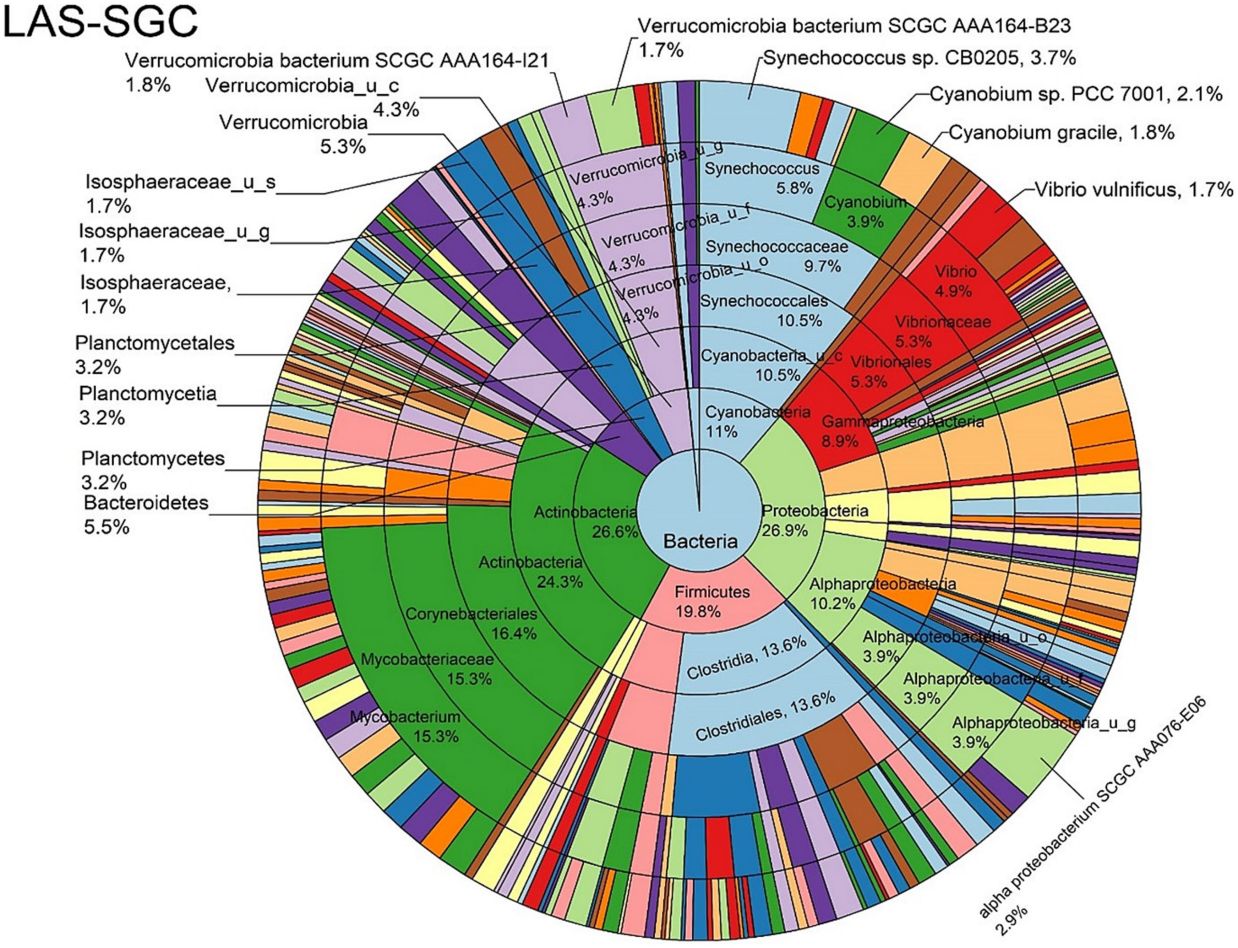
Log abundance distribution of bacterial taxa in the SGC samples. LAS, log abundance score; SGC, Stomach gut contents.

**Figure 9:**
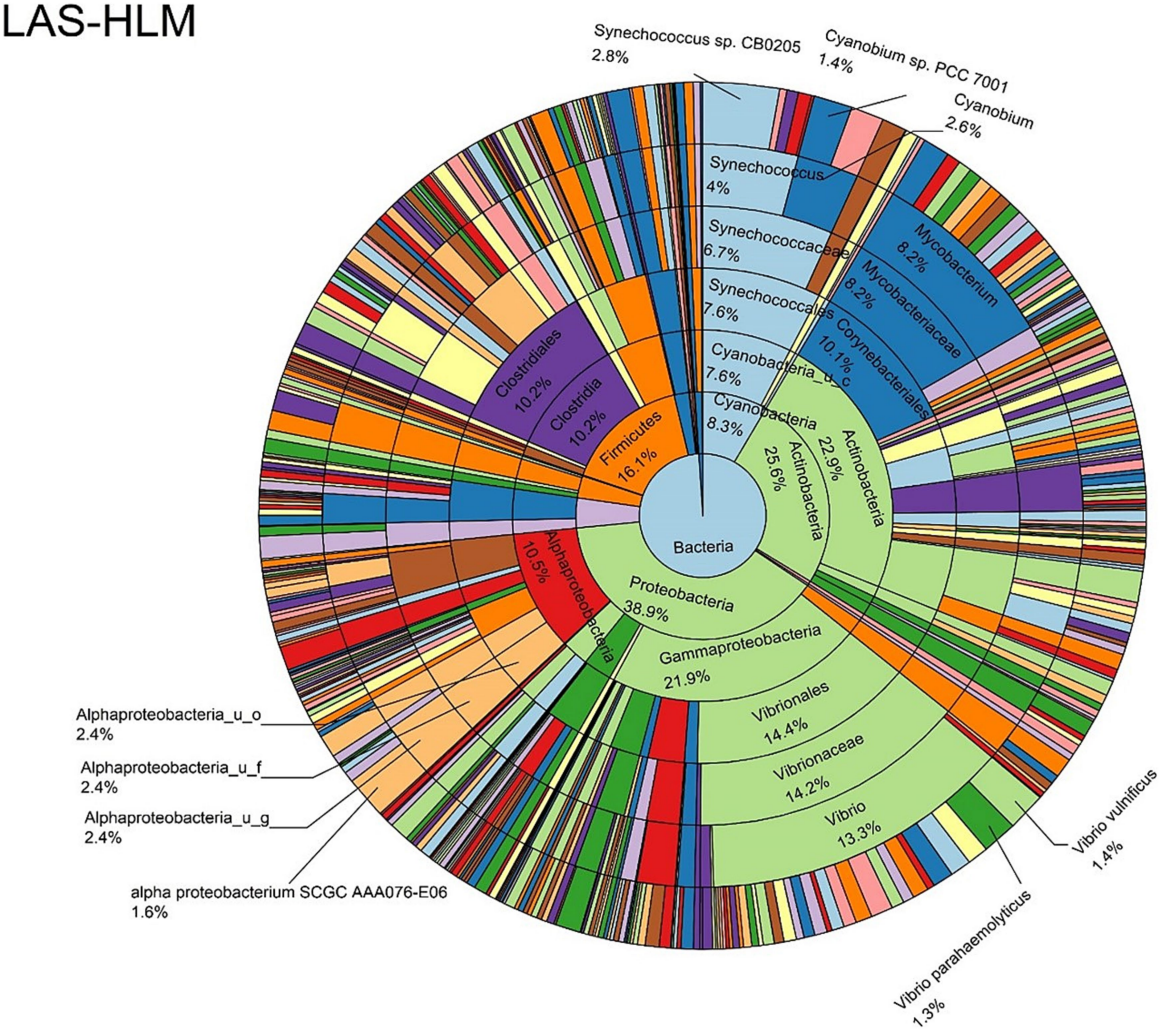
Log abundance distribution of bacterial taxa in the HLM samples. LAS, log abundance score; HLM, Oyster-hemolymph.

The log abundance score of the sequences obtained from this study revealed that the top six dominant bacterial species that had the highest mean abundance and ubiquity across all sample types were *Synechococcus* sp. CB0205, *Vibrio vulnificus*, *alpha proteobacterium* SCGC AAA076-E06, *Cyanobium* sp. PCC 7001, *Vibrio parahaemolyticus*, and *Cyanobium gracile*, respectively (Figure 2). On a per-sample type basis, *Synechococcus* sp. CB0205, had the highest relative log abundance in the DSH, SGC, and HLM; *Vibrio vulnificus* in the AOH, EOH, and DSH; and *Cyanobium* sp. PCC 7001 in the FOH (Figures 3–9). *Vibrio parahaemolyticus* and *alpha proteobacterium* SCGC AAA076-E06 had the second highest relative log abundance in the EOH and SGC, respectively (Figure 3). *Synechococcus* sp. CB0205 and *Vibrio vulnificus* ranked, in most cases, among the top five species in all sample types (Figure 3). However, the abundance score indicated that FOH, SGC, and HLM samples were dominated by *Synechococcus* sp. CB0205, AOH, and DSH were dominated by *Vibrio vulnificus*, and EOH was dominated by *Photobacterium damselae* (Supplementary Figures S2–S8). Based on the abundance and log abundance scores, *Synechococcus* sp. CB0205 consistently emerged as the dominant species across most sample types (Figures 4–9; Supplementary Figures S3–S8). However, when considering the log abundance score, other species like *Vibrio vulnificus*, *Photobacterium damselae*, and *Cyanobium* sp. PCC 7001 also exhibited high relative abundances (Figures 4–9). These findings suggest that *Synechococcus* sp. CB0205 is a prevalent and ubiquitous microorganism in oysters, while other species showed variable dominance depending on the sample type.

#### Diversity of bacterial species

**Figure 10:**
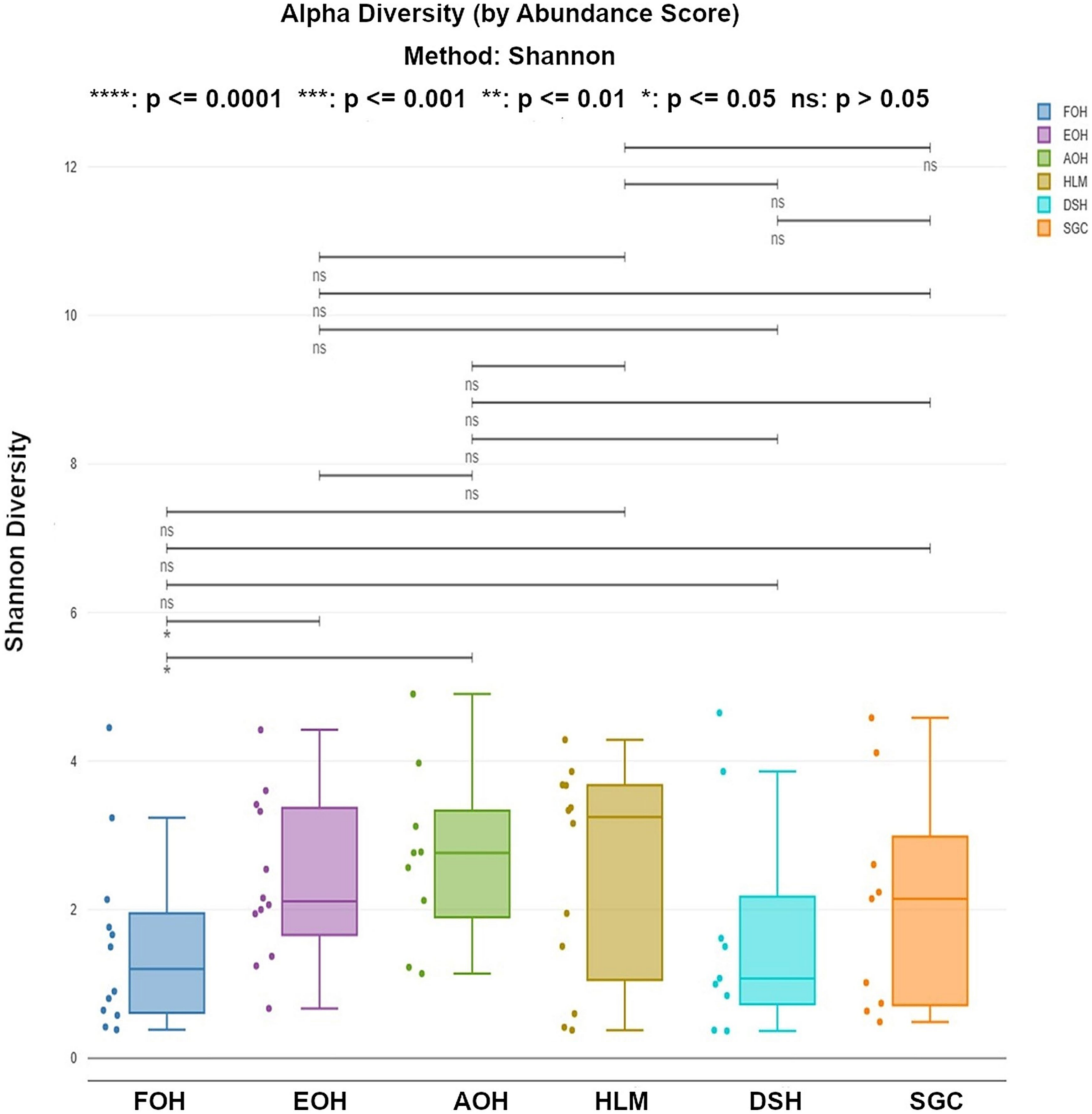
Shannon index representing the richness and evenness of bacterial species within all sample types. Each dot represents the Shannon diversity value for an individual sample. Statistical comparisons of medians across sample types were performed using a Wilcoxon rank-sum test. FOH, Fresh-oyster homogenate; AOH, Temperature abused-oyster homogenate; EOH, Enriched-oyster homogenate; DSH, Dissected stomach homogenate; SGC, Stomach gut contents; HLM, Oyster-hemolymph.

The alpha diversity of bacterial species was assessed using the Shannon index, which provides information about both richness and evenness. The median of Shannon values ranged from 1.1 in the DSH to 3.2 in the HLM (Figure 10). The richness and evenness were significantly higher in the AOH and EOH than in FOH, as indicated by the Wilcoxon Rank Sum test (*p* < 0.05) (Figure 10; Supplementary material). The Bray-Curtis index was used to assess the differences in microbial composition and their relative abundance (beta diversity) between the sample types. The results showed that the EOH had the most distinct microbial composition and relative abundance compared to all other sample types, as determined by the PERMANOVA test (*p* < 0.05) (Table 3).

**Table 3:** Significant differences in bacterial composition (Bray-Curtis) within all sample types.

Feature	Sample type	Statistic	*p*-value
Bacteria	FOH ↔ EOH	3.482	0.001
	AOH ↔ EOH	1.971	0.035
	EOH ↔ DSH	2.721	0.004
	EOH ↔ SGC	3.597	0.004
	EOH ↔ HLM	2.603	0.011
Phages	FOH ↔ AOH	17.42	0.001
	FOH ↔ EOH	1.982	0.044
	AOH ↔ EOH	7.418	0.001
	AOH ↔ DSH	10.616	0.001
	AOH ↔ SGC	10.491	0.001
	AOH ↔ HLM	6.842	0.005
Viruses	FOH ↔ AOH	3.004	0.004
	AOH ↔ EOH	2.467	0.015
	AOH ↔ DSH	3.583	0.005
	AOH ↔ SGC	4.43	0.003
	AOH ↔ HLM	2.554	0.035
Fungi	FOH ↔ AOH	4.032	0.02
	AOH ↔ SGC	3.431	0.029
Protist	FOH ↔ AOH	3.892	0.027
	FOH ↔ EOH	2.401	0.028
	FOH ↔ HLM	2.526	0.023
	AOH ↔ EOH	2.676	0.006

FOH, Fresh-oyster homogenate; AOH, Temperature abused-oyster homogenate; EOH, Enriched-oyster homogenate; DSH, Dissected stomach homogenate; SGC, Stomach gut contents; HLM, Oyster-hemolymph.

#### Differential bacterial taxonomic composition between sample types

Significant differences in the oyster microbiome were observed between sample types. Thirty-six differential bacterial taxa (LEfSe: LDA score > 2) were identified to be the taxonomic signature of a particular sample type (Supplementary Figure S9). For example, at the bacterial species level, *Cyanobium* sp. PCC 7001, *Pantoea septica*, and *Cyanobium gracile*; *Escherichia coli*, *Parabacteroides distasonis*, and *Eggerthella* sp. HGA1; *Photobacterium damselae, Vibrio parahaemolyticus*, and *V. vulnificus*; *V. diazotrophicus*, *Brevundimonas* sp. and *Dermacoccus nishinomiyaensis*; alpha proteobacterium SCGC AAA160-J14; *Pelagibacteraceae* sp. and *Micrococcus luteus* were the most distinctive bacterial species in the FOH, AOH, EOH, DSH, SGC, and HLM samples, respectively. *Synechococcus* sp. CB0205 was highly abundant in all sample types, which may explain why it wasn’t a distinctive feature of any sample type (Supplementary Figure S10). Moreover, among the top abundant species, *Vibrio vulnificus* and *V. parahaemolyticus* demonstrated a significantly higher prevalence in the EOH samples compared to the majority of other sample types (Supplementary Figures S11, S12).

#### Abundance and ubiquity of viruses, fungi, and protists among sample types

In addition to bacterial taxa identification, raw sequencing reads were interrogated to identify other microbial organisms, namely DNA viruses and prophages, fungi, and protists. The abundance of bacterial sequences exceeds that of other classified sequences, as evidenced by the ratios of phage, viral, protist, and fungal sequences to bacterial sequences, which are 1:10, 1:97, 1:125, and 1:246, respectively. Viruses that had the highest mean log-abundance score within all sample types included marine snail associated circular virus, *Chrysochromulina ericina* virus, *Circoviridae* 10 LDMD-2013, tomato brown rugose fruit virus, and *Rosellinia necatrix* partitivirus 8, respectively (Supplementary Figure S13). As per each sample type, *Chrysochromulina ericina* virus was the dominant virus of the FOH and DSH, while marine snail associated circular virus was the dominant virus in the AOH, EOH, SGC, and HLM as demonstrated by the relative log-abundance (Supplementary Figure S14).

The dominant phages within all sample types were *Synechococcus* phage S-CBS1, *Cyanophage* KBS-S-2A, *Synechococcus* phage S-CBP4, *Synechococcus* phage S-CBP1, *Synechococcus* phage S-CBP3, respectively (Supplementary Figure S15). Phages with the highest relative log-abundance within each sample type include *Myoviridae* sp. in the FOH and DSH; *Escherichia* virus HK630 in the AOH; *Cyanophage* KBS-S-2A in the EOH and SGC; *Synechococcus* phage S-CBP1 in the HLM (Supplementary Figure S16).

The protists and fungi identified in this study and their abundance were remarkably lower than the other microbial species in all sample types. Dominant protist species within all sample types were *Entamoeba hartmanni*, *Thalassiosira* sp., *Salpingoeca rosetta*, *Chromera velia*, respectively (Supplementary Figure S17). Furthermore, these species were the dominant species in the AOH/SGC, EOH/HLM, FOH, and DSH, respectively (Supplementary Figure S18). The dominant fungi species within all sample types were *Agaricomycetes* sp., *Brettanomyces bruxellensis*, *Malassezia restricta*, *Onygenales* sp., and *Malassezia globosa*, respectively (Supplementary Figure S19). However, *Agaricomycetes* sp. was the dominant fungi in the EOH, DSH, SGC, and HLM, while *Malassezia restricta* and *Fungi* sp. were the dominant in the FOH and AOH, respectively (Supplementary Figure S20).

#### Diversity of viruses, fungi, and protists

The median of the Shannon values of the viruses ranged from 0.4 in the SGC samples to 1.6 in the DSH samples (Supplementary Figure S21), while for the phages it ranged from 1.5 in the AOH samples to 3.7 in the EOH samples (Supplementary Figure S22). The Wilcoxon Rank Sum test revealed that the diversity of viruses in the SGC sample was significantly lower compared to the FOH, AOH, and DSH samples (Supplementary Figure S21). Phages in the AOH samples were significantly lower than all other sample types (Supplementary Figure S22). Due to their low abundance, the statistical analysis of the Shannon values for the protist and fungi between the sample types could not be calculated.

The Bray-Curtis index and PERMANOVA analysis revealed significant differences in the composition and relative abundance (beta diversity) of viruses and phages in the AOH samples compared to all other sample types (Table 3). Additionally, the phages in the EOH sample exhibited significant differences compared to those in the FOH samples. The composition and relative abundance of protists in the FOH samples showed significant differences compared to those in the AOH, EOH, and HLM samples (Table 3). Furthermore, the protists in the AOH samples were significantly different from those in the EOH samples. Fungi composition and relative abundance in the AOH sample were significantly different from those in the FOH and SGC samples (Table 3).

#### Differential phage and viral taxonomic composition between sample types

A total of thirty-two phage taxa exhibiting significant differences (LEfSe: LDA score > 2) were identified as distinct features specific to each sample type (Supplementary Figure S23). Among these phages, *Synechococcus* phage S-CBP3, *Synechococcus* phage S-CBP1, and *Synechococcus* phage S-RIM8 A.HR1; *Escherichia* virus HK630, *Enterobacteria* phage SfI, and *Enterobacteria* phage HK140; *Vibrio* virus pVp1, *Vibrio* virus VfO3K6, and *Escherichia phage* TL-2011b; *Vibrio* phage martha 12B12, *Synechococcus* phage S-PM2, and Autographiviridae sp.; *Synechococcus* phage S-CBP4, *Synechococcus* phage S-RIP1, and *Synechococcus* virus P60; *Cyanophage* S-RIM50, *Synechococcus* phage S-WAM2, and *Synechococcus* phage S-RSM4 were the distinctive phages in the FOH, AOH, EOH, DSH, SGC, and HLM, respectively. Additionally, a set of 14 viral taxa with significant distinctions (LEfSe: LDA score > 2) were identified as characteristic features of specific sample types (Supplementary Figure S24). Notably, piscine myocarditis-like virus, *Penicillium chrysogenum* virus, *Nyamanini nyavirus*, and *Cardiovirus* A; thrips-associated genomovirus 2, *Salmonella* virus ST64T, and *Shigella* virus Sf6; *Vibrio* virus Vf33, *Micromonas* sp. RCC1109 virus MpV1, and *Micromonas pusilla* virus 12T; *Chrysochromulina ericina* virus were the most distinctive viruses of the FOH, AOH, EOH, and SGC, respectively.

#### Antimicrobial resistance genes and virulence factors

A total of 91 unique antimicrobial resistance genes (ARGs) were identified in all sample types (Supplementary material). The total abundance of the ARGs in each sample type ranged from (>3.5 k) from the SGC to (>1.5 m) from the EOH (Table 4). All the ARGs identified in this study belong to 33 drug classes, of which 18 (39 genes) were only identified in the EOH. Of these 39 gene sequences, 20 were related to six classes of multi-drug resistance (MDR).

**Table 4:** Number of biomarkers identified and their abundance score among sample types.

Processing method	Bacteria (AS)	Phages (AS)	Viruses (AS)	Fungi (AS)	Protists (AS)	ARGs (AS)	FVGs (AS)
FOH	274 (623,980)	99 (267,313)	33 (19,905)	10 (280)	28 (200)	9 (13,527)	23 (66,859)
AOH	175 (82,086)	62 (16,801,964)	35 (246,872)	2 (21)	3 (581)	7 (26,095)	18 (822,220)
EOH	476 (2,258,615)	183 (3,882,483)	43 (5,359,925)	10 (1,375)	16 (879)	68 (1,565,101)	52 (435,092)
DSH	314 (2,064,250)	108 (138,994)	31 (18,824)	7 (159)	12 (187)	11 (20,170)	26 (55,793)
SGC	224 (379,111)	114 (841,342)	30 (646,798)	4 (118)	9 (345)	4 (3,596)	12 (33,369)
HLM	400 (1,085,763)	148 (6,237,708)	45 (3,674,373)	14 (445)	16 (1,828)	7 (7,928)	31 (154,139)

AS, abundance score; ARGs, antibiotic resistant genes; VFGs, virulence factor genes; FOH, Fresh-oyster homogenate; AOH, Temperature abused-oyster homogenate; EOH, Enriched-oyster homogenate; DSH, Dissected stomach homogenate; SGC, Stomach gut contents; HLM, Oyster-hemolymph.

Beta-lactam-resistance genes (*^bla^* CARB 23, *^bla^* CARB 21, and *^bla^* CARB 17) had the highest log mean abundance within all sample types (Supplementary Figure S25). However, On a per-sample type basis, Aminoglycoside *aadD*, Tetracycline *tetC*, Beta-lactam-resistance *^bla^* CARB 23, Aminoglycoside *aph3’* III, Beta-lactam-resistance *^bla^* OXA 264, and Beta-lactam-resistance genes *^bla^* CARB 23, 21, and 17 were the dominant in the FOH, AOH, DSH, SGC, HLM, and EOH, respectively, (Supplementary Figure S26).

On the other hand, 133 unique virulence factor genes (VFGs) were identified in all sample types (Supplementary material). The total abundance of the VFGs in each sample type ranged from (>33 k) from the SGC to (>822 k) from the AOH (Table 4). Twinty-four bacterial taxa were associated with these VFGs, and most of these genes (97) were associated with *Escherichia coli*, *Francisella tularensis*, *Enterobacter aerogenes*, *Enterobacter*, *Bacteroides fragilis*, and *Enterococcus faecalis*, respectively (Supplementary material). The presence of *Staphylococcus lentus* genes *ermC* and *repL* was notably prominent exclusively in the FOH samples, indicating their frequent detection solely within this specific sample type (Supplementary Figure S27). *Escherichia coli* GENE *iss* had the highest log mean abundance across all sample types (Supplementary Figures S27, S28).

The statistical analysis of the Shannon values for the ARGs and VFGs between the sample types could not be calculated. On the other hand, Bray-Curtis values indicated that the composition and the relative abundance of the ARGs and VFGs in the EOH and FOH were significantly distinct from all sample types, respectively (Supplementary material).

## Discussion

This study comprehensively examined microbiome profiles of the eastern oyster using shotgun metagenomic sequencing of different specimen types and processing conditions of individual oysters from the Chesapeake Bay. Compared to previously reported studies of the eastern oyster microbiome, this study included a larger number of oyster replicates and sampling time. The oyster’s microbiome was analyzed at the species level which provided more details about the microbial composition and their relative abundance, antimicrobial resistance genes, and the virulence factors associated with each microbial community.

In this study, we utilized saponin treatment to reduce the host DNA background and improve microbial DNA representation for shotgun metagenomic sequencing. Saponin treatment has been successfully applied to various sample types, including human and food samples (Bloomfield et al., 2023; Hasan et al., 2016). Despite its intended purpose, the saponin treatment in our study did not effectively reduce the proportion of host DNA, as indicated by the ratio of raw reads to microbial hits (Table 2), at an expected level. That said, we cannot fully conclude its efficacy without a direct comparison to untreated samples. Future studies could include such comparisons to better assess the relative effectiveness of saponin in this context.

Nevertheless, the results of our analysis indicate that saponin treatment did not affect the biological representativeness of certain bacterial genera commonly found in the eastern oyster microbiome. These included *Synechococcus*, *Cyanobium*, *Vibrio*, *Photobacterium*, *Escherichia*, *Shewanella*, *Francisella*, *Mycobacterium*, and *Bacteroides*, belonging to the phyla Cyanobacteria, Proteobacteria, Actinobacteria, and Bacteroidetes, respectively. The relative abundance of these phyla in our study was relatively high across various sample types, which aligns with findings from previous studies (Chauhan et al., 2014; Hines et al., 2023; King et al., 2012; Pathak et al., 2023; Pierce and Ward, 2018, 2019; Pimentel et al., 2020, 2021). Additionally, the relative abundance of other phyla such as Firmicutes was relatively high in some samples, which is also consistent with previous findings (Hines et al., 2023; King et al., 2012; Pierce and Ward, 2018, 2019), even though their constituent genera did not comprise a large percentage compared to the aforementioned genera. However, other classes/genera and their corresponding phyla, such as Mollicutes (Mycoplasmatota), Chlamydiae (Chlamydiota), Spirochaetia (Spirochaetota), and Fusobacteriia (Fusobacteriota), as well as the phylum Verrucomicrobia, which have been reported as dominant in previous eastern oyster microbiome studies (Hines et al., 2023; King et al., 2012; Pierce and Ward, 2018, 2019; Pimentel et al., 2020, 2021), were either not detected or present at extremely low levels in our findings. These discrepancies may be attributed to biases inherent in the sequencing methods, such as the specific primer combinations used in 16S rRNA sequencing, which target different variable regions (e.g., V1–V2, V4–V5) (Clooney et al., 2016; Logares et al., 2014; Tessler et al., 2017). These primer choices can influence the detection of certain taxa. Additionally, PCR amplification in 16S rRNA sequencing can introduce biases, preferentially amplifying some taxa over others (Clooney et al., 2016; Logares et al., 2014; Tessler et al., 2017).

### Bacterial diversity and composition among sample types

The percentage of bacterial hits was higher compared to other microbial hits identified in this study and varied across sample types. The variation in bacterial hit percentages among sample types reflects the impact of processing on microbial DNA recovery. Enrichment of EOH samples led to the highest bacterial hit percentage, demonstrating the enrichment’s effectiveness in enhancing bacterial DNA representation. Conversely, AOH samples had the lowest bacterial hit percentage, which may be attributed to the effects of temperature abuse on microbial recovery. These results underscore how handling and processing factors influence the load of bacterial DNA retrieved from different sample types.

The log relative abundance of bacterial species also differed across sample types, with certain species dominating specific samples. For example, *Synechococcus* sp. CB0205, *Vibrio vulnificus*, and *Cyanobium* sp. PCC 7001 were consistently among the top species in various sample types, indicating their ecological importance. Comparisons with previous studies indicate that the prevalence and ubiquity of these species are consistent findings among the bacterial community of the eastern oysters and the marine environment of the Chesapeake Bay (Cai et al., 2010; Parveen et al., 2020; Wang et al., 2020). However, the specific dominant species varied among the sample types. This suggests that different oyster sample types may selectively enrich or support the growth of specific bacterial taxa, potentially influenced by factors associated with each sample type.

The alpha diversity, as measured by the Shannon index, varied among the sample types. Statistical analysis showed that Shannon values of the AOH and EOH samples were significantly higher in richness and evenness compared to FOH. This indicates that processing conditions can affect bacterial richness and evenness in oysters, suggesting that these sample types may provide a more representative snapshot of the overall microbial diversity associated with oysters.

The beta diversity, assessed using the Bray-Curtis index, indicated that the microbial composition and their relative abundance of EOH were significantly distinct from other sample types. This indicates that the enrichment process significantly influenced the microbial community composition in EOH samples. The distinctiveness of EOH further emphasizes the importance of considering these processing conditions when investigating the oyster microbiome. Even though the enrichment process may introduce biases and selectively promote the growth of certain bacterial species, including mesophilic bacteria like *Vibrio*, the taxa identified in this study were similar among sample types. Taxa that were not identified or were at extremely low levels compared to previous studies were not just associated with the enriched samples but also with the other sample types. It is likely that the enrichment time (10 h) used in this study was sufficient to improve the representation of microbial biomass without introducing significant biases against certain taxa.

LEfSe analysis indicated that 36 bacterial taxa were the characteristic of specific sample types. For instance, *Cyanobium* sp. PCC 7001 and *Pantoea septica* were the significant features in FOH samples, while *Vibrio vulnificus* and *V. parahaemolyticus* were the significant features in EOH samples. *Synechococcus* sp. CB0205 was abundant across all sample types, which may prevented it from being identified as a significant feature of any specific group. These sample-type-specific microbial signatures may have contributed to the observed diversity differences.

### Viruses, fungi, and protist diversity and composition among sample types

The abundance and diversity of viruses were examined, and the findings highlight the variation in viral composition among the sample types. *Chrysochromulina ericina* virus and marine snail associated circular viruses were consistently identified across multiple sample types in the present study, suggesting their potential association with oyster viromes. Among the identified viruses, Human polyomavirus 2 (HPyV2) is of particular concern due to its association with neurological disease in susceptible individuals (Abreu et al., 2020; Vago et al., 1996). HPyV2 has been detected in oyster samples and marine environments, and due to its persistence, it can be used as an indicator of contamination (Abreu et al., 2020; Rachmadi et al., 2016). However, it is important to note that HPyV2 was detected in all sample types and may temporarily contaminate the environment of the oyster as they were only identified in the oyster replicates collected in May and June. Plant viruses belonging to the genera *Tobamovirus* were also detected in the oyster samples, including EOH, SGC, and HLM. Although highly abundant in oyster samples, there was no evidence to suggest that these plant viruses replicate within oysters. The presence of plant viruses may be attributed to temporary contamination from the surrounding environment, potentially through infected plants growing near the water, plant material, or sewage (Mehle and Ravnikar, 2012).

Shannon analysis demonstrated that virus diversity in SGC was significantly lower than FOH, AOH, and DSH. The AOH samples displayed significant differences in viral composition and relative abundance compared to all other sample types. This suggests that AOH samples may provide a distinct representation of the oyster-associated viral community.

In all sample types, the number of phages identified generally corresponded to the number of bacterial species identified; however, phage abundance scores did not show the same correspondence and varied notably between sample types. This highlights complex ecological interactions in the oyster microbiome. The abundance score of phages in the AOH, EOH, SGC, and HLM was higher than bacterial species. These sample types may have had a higher competitive environment in which the cost of persistence largely affected the densities of bacterial species (Harcombe and Bull, 2005). Another interpretation would be that these sample types contain more multi-host phages or multi-phage bacterial species than the other sample types (Comeau et al., 2005). The number of phages identified in the SGC was slightly higher than in the DSH, and the abundance score in the SGC was notably higher, indicating that tissue-free specimens may enhance the detection and representation of phages.

Phages that had the highest mean log abundance across all sample types were *Synechococcus* phage S-CBS1, *Cyanophage* KBS-S-2A, *Synechococcus* phage S-CBP4, *Synechococcus* phage S-CBP1, and *Synechococcus* phage S-CBP3. Although the most dominant *Synechococcus* phages (9 phages) belong to *Siphoviridae* and *Autographiviridae* families, most of the *Synechococcus* phages (35 phages) identified in all sample types belong to *Myoviridae* family. This is not in contrast to what has been reported, since the vast majority of phages recovered from *Synechococcus* strains belong to *Myoviridae* (Clokie and Mann, 2006). *Synechococcus* phages are known to infect *Synechococcus* bacteria which was among the most abundant bacterial species in all sample types (Clokie et al., 2011), indicating their association with oyster viromes. Most of the phages associated with *Vibrio* were found to have high log abundance in the EOH samples, indicating their potential association with *Vibrio* abundance. *Vibrio* virus pVp1, *Vibrio* virus VfO3K6, and *Vibrio* phage PV94 were among the most distinctive *Vibrio* phages of the EOH samples. This suggests that *Vibrio* abundance in EOH may significantly influence the abundance of *Vibrio* phages, as phages thrive in environments where their bacterial hosts proliferate (Clokie et al., 2011).

Phages taxa identified in the AOH were the lowest in contrast to their total abundance score, which was the highest, compared to the other sample types. This agrees with the phages richness and evenness values (alpha diversity) which were significantly lower than all sample types. Phages composition and their relative abundance (beta diversity) in the AOH were also significantly distinct from all sample types. This indicates that temperature abuse can significantly impact the alpha and beta diversity of the phage community in the eastern oyster.

Our findings indicated that the oyster viromes were predominantly composed of families from the order Caudovirales, specifically *Siphoviridae*, *Myoviridae*, *Autographiviridae*, and *Podoviridae* (Supplementary material). We also observed families outside of Caudovirales, such as *Circoviridae* and *Virgaviridae*, which were prominent in the oyster viromes. These findings align with previous reports, underscoring the diversity of viral families within oysters and confirming the consistency of our results with existing literature on oyster viromes (Dupont et al., 2020; Jiang et al., 2023).

Protist and fungal sequences exhibited remarkably lower abundances, suggesting that protists and fungi may have limited diversity within the oyster microbiome compared to bacteria and viruses. This can be attributed to different factors including their natural abundance in the environment, accumulation rates in oysters, or biases in sample preparation and DNA extraction. Among the oyster sample types, the FOH samples appear to better represent the protist and fungal community as they capture a more representative snapshot of its diversity within oysters.

*Acanthamoeba polyphaga* was mostly detected in FOH, and it was one of its most distinctive species. Although protists can contribute to bacterial reduction as a result of grazing, some species such as *Acanthamoeba polyphaga*, raise concerns as they have been demonstrated to act as hosts for pathogens, potentially contributing to the persistence of pathogenic bacteria in the environment (Vaerewijck et al., 2014). It has been reported that some *Vibrio* species including *V. parahaemolyticus* and *V. cholerae* can survive and multiply when cocultured with *Acanthamoeba castellanii* and *A. polyphaga*, respectively (Vaerewijck et al., 2014). The organic matter and moisture-rich environment of oysters may provide an ideal habitat for protists, this raises the concern of the possibility of a positive correlation between pathogens and free-living protists.

It is worth noting that protists that can cause oyster diseases, such as *Haplosporidium nelsoni*, responsible for multinucleate sphere X, and *Bonamia* spp., causing bonamiasis, were not detected in any of the sample types. However, a scarce level of *Perkinsus marinus*, responsible for Dermo disease, was detected in FOH, AOH, EOH, DSH, and HLM, mostly in one replicate of each sample type. Apparently, the presence of *Perkinsus marinus* did not indicate any signs of disease within these replicates, as it did not result in any unusual outcomes in the microbiome profiling of these replicates.

### ARGs and VFGs diversity and composition among sample types

The detection of antimicrobial resistance genes (ARGs) in the oyster sample types was generally very low, except for the replicates obtained from the EOH. This observation aligns with previous reports indicating that quasimetagenomic methods may offer improved sensitivity for ARGs detection compared to traditional metagenomic techniques (Ottesen et al., 2022). The EOH replicates displayed a slightly higher abundance of ARGs, particularly those collected during the months of August and September. Notably, one EOH replicate collected in August exhibited the detection of 52 out of the 91 ARGs identified across all sample types, with 39 of these ARGs exclusively detected in this particular replicate. Moreover, a majority of the multidrug-resistant genes were identified in this replicate. This observation can be attributed to the presence of *Photobacterium damselae* subsp. *damselae* CIP 102761 (pdd) strain. The abundance of this specific strain in that particular replicate accounted for approximately 76% of its abundance across all sample types. Furthermore, it was the most abundant strain within this replicate, contributing to 92% of the total abundance score of all species present. It has been reported that pdd harbors a conjugative MDR plasmid dubbed pAQU1 (Vences et al., 2020). Plasmids related to pAQU1 were reported in *Vibrio* species (Vences et al., 2020), which may also explain the high abundance of MDR in the EOH. Beta analysis of ARGs indicated that, except for the EOH, no significant differences were found between the FOH and the remaining sample types. This may indicate that ARGs are persistent in the aquatic environment of the oysters analyzed in this study, and the abundance of ARGs was not tissue specific. Beta diversity of the EOH showed that ARGs were significantly distinct from all sample types, while FVGs were significantly distinct from the FOH and AOH. These findings suggest that enrichment could affect the growth of antibiotic resistant pathogenic bacterial species.

Like the ARGs, VFGs identified in all sample types of oysters were relatively low. However, specific VFGs exhibited noteworthy patterns across different sample types. For instance, *Staphylococcus lentus* genes *ermC* and *repL* were prominently detected exclusively in the FOH samples. Intriguingly, the presence of *S. lentus* species was not observed in the FOH samples, and instead, only *S. epidermidis* was identified in the FOH replicates that displayed *ermC* and *repL* genes. The taxonomic identification of *S. sciuri* group members, to which *S. lentus* belongs, has posed challenges, leading to continuous taxonomic revisions (Nemeghaire et al., 2014). Notably, certain virulence and antimicrobial genes previously reported in the *S. sciuri* species group were predominantly found in other *Staphylococci*. This suggests that the *S. sciuri* group serves as a substantial reservoir of exchangeable genes for other *staphylococci* and bacterial species (Nemeghaire et al., 2014). In contrast, the AOH replicates exhibited a high abundance of *Escherichia coli*, coinciding with the elevated presence of the *Escherichia coli* gene *iss*. The gene *iss* is known to be associated with extraintestinal pathogenic *E. coli* (ExPEC) strains and is less prevalent among other *E. coli* pathotypes and human fecal commensal *E. coli* (Johnson et al., 2008). The abundance of *E. coli* in the AOH replicates, coupled with the high prevalence of the *iss* gene, suggests a potential association with non-fecal or non-human *E. coli* strains.

## Conclusion

This study provides valuable insights into the oyster microbiome under different specimen types and processing conditions, analyzed using shotgun metagenomic sequencing. Our findings highlight the heterogeneity of microbial communities and the influence of specimen type and processing on microbial composition. The results also revealed potential limitations of saponin treatment for reducing host DNA, suggesting a need for improved methods to optimize metagenomic studies.

Notably, enriched oyster samples (EOH) exhibited higher bacterial hits, distinct microbial profiles, and elevated reads for bacterial species, phages, and antimicrobial resistance genes. While EOH processing may be useful for targeting specific microbial groups, our results emphasize the importance of integrating enriched and non-enriched methods to achieve a comprehensive understanding of the oyster microbiome.

Understanding the variation in bacterial communities can aid in optimizing protocols for food safety testing and identifying microbial signatures associated with oyster health. Future research should focus on refining processing methods, reducing host DNA interference, expanding sample sizes, and incorporating shorter collection intervals to capture the broader diversity of oyster-associated microbial communities. Additionally, investigating the potential implications of the identified dominant bacterial species, including their roles in oyster health and disease, could provide valuable insights for oyster risk management.

## Data Availability

Raw sequence reads have been deposited in the NCBI database under BioProject number PRJNA1036668, accession numbers SAMN38147165 to SAMN38147227.
