# Supplementary material for: Comparative evaluation of specimen type and processing conditions for studying oyster microbiomes
Source: Front Microbiol. 2025 Jan 8;15:1504487. doi: 10.3389/fmicb.2024.1504487 (PMC11750828; doi:10.3389/fmicb.2024.1504487)
Supplement: Supplementary file 1 [file Data_Sheet_1.pdf]

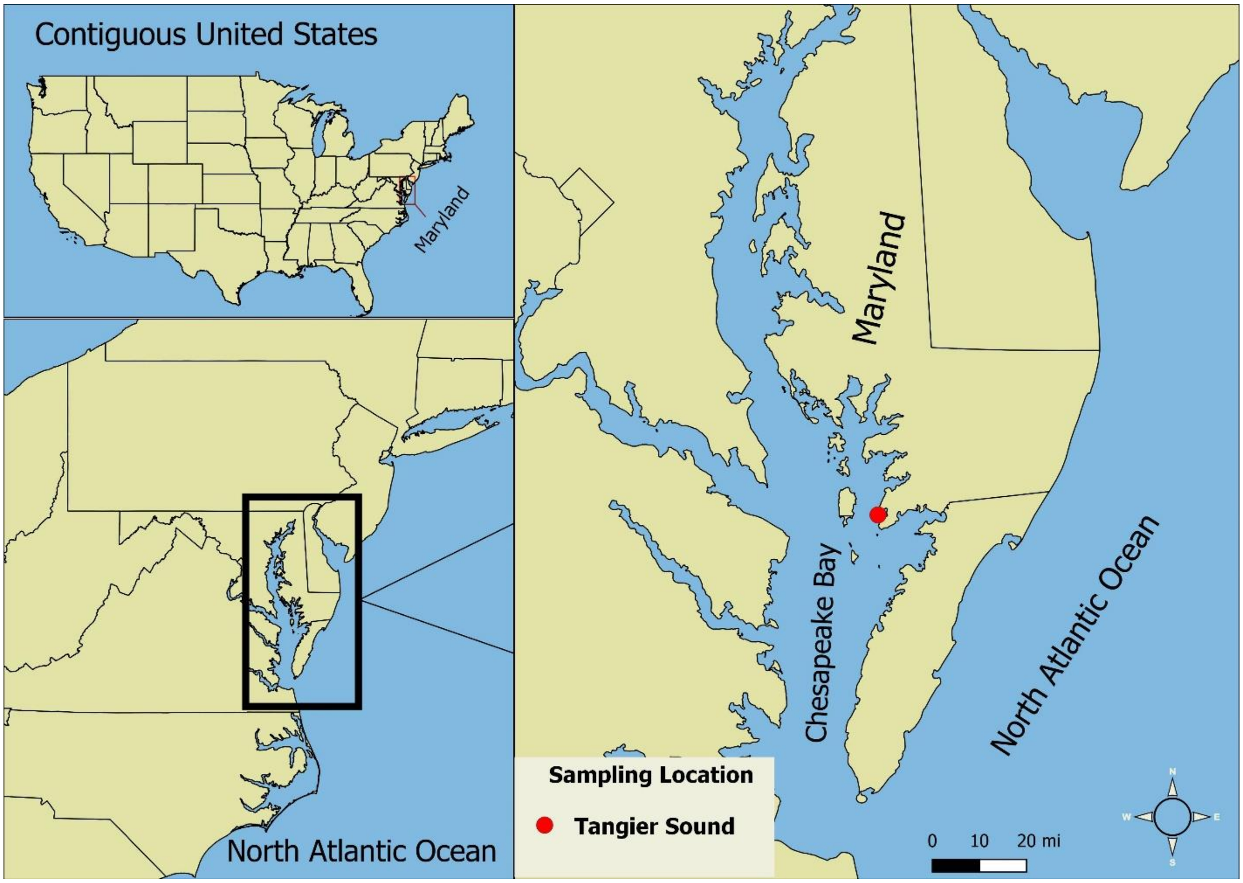

Figure S1**:** Map of the Chesapeake Bay sampling site.

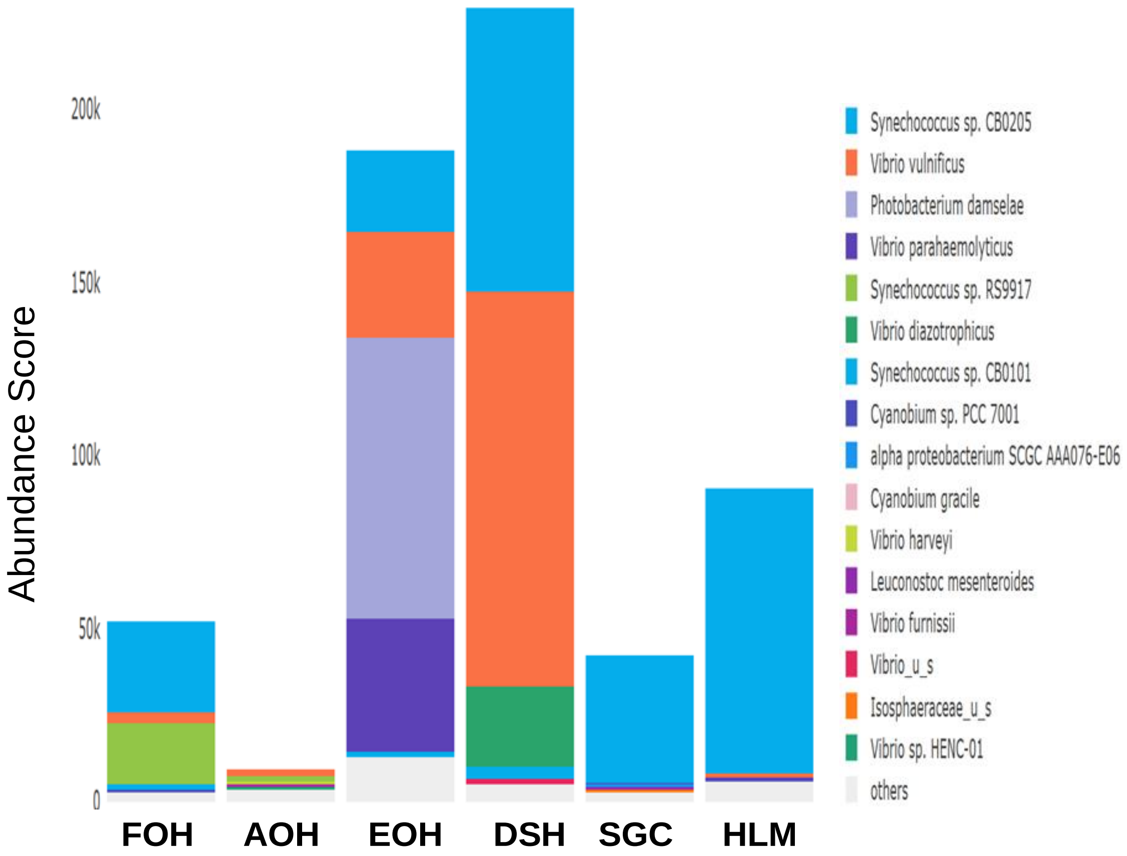

Figure S2**:** Relative abundance score of bacterial taxa in relation to sample type.

FOH: Fresh-oyster homogenate; AOH: Temperature abused-oyster homogenate; EOH: Enriched-oyster homogenate; DSH: Dissected stomach homogenate; SGC: Stomach gut contents; HLM: Oyster-hemolymph.

AS-FOH

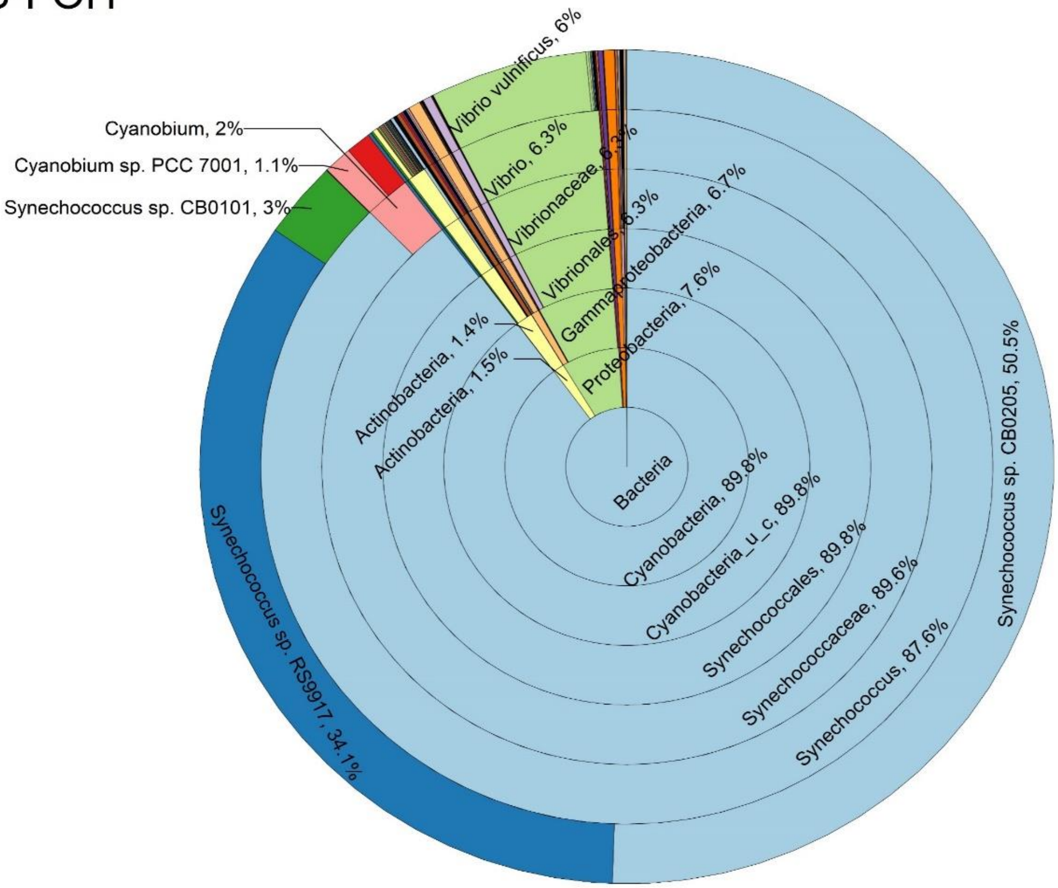

Figure S3**:** Abundance distribution of bacterial taxa in the FOH samples. AS: abundance score. FOH: Fresh-oyster homogenate.

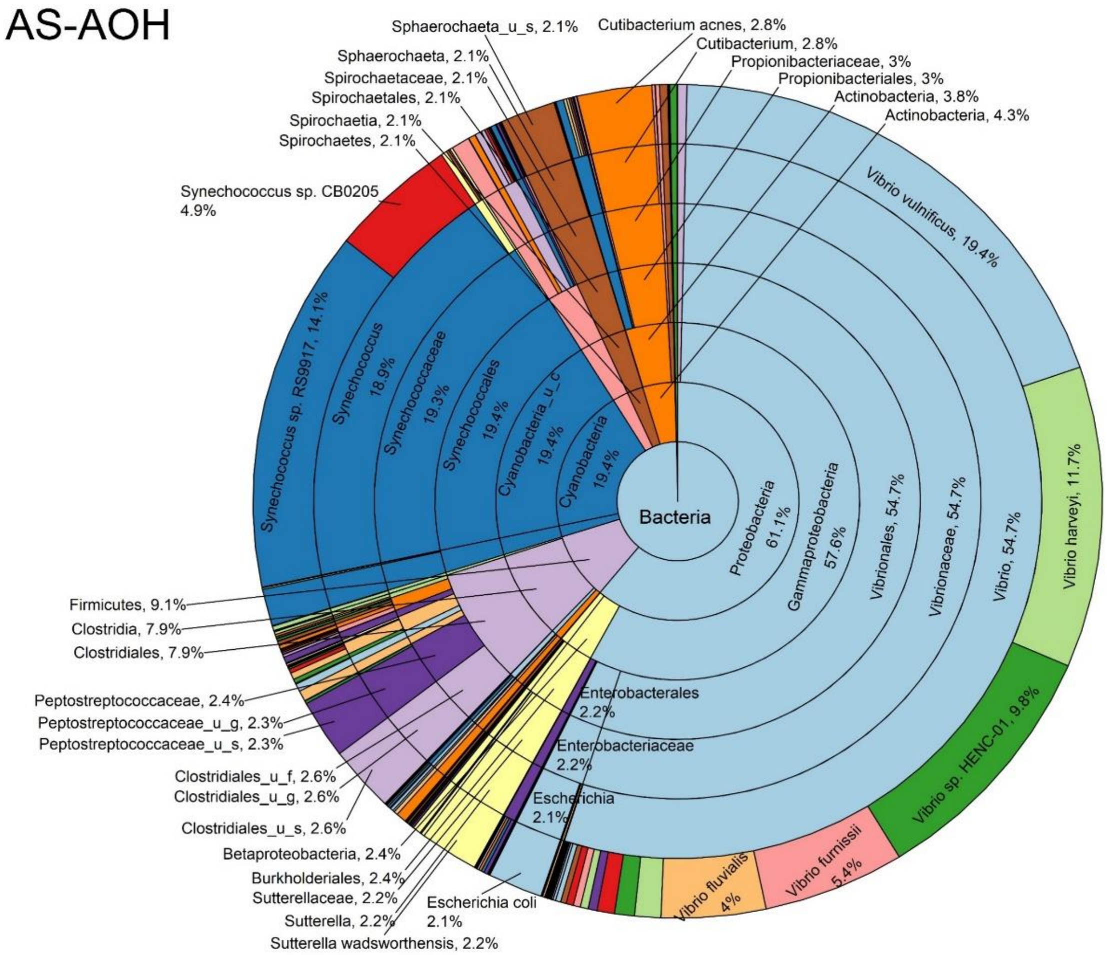

Figure S4**:** Abundance distribution of bacterial taxa in the AOH samples. AS: abundance score. AOH: Temperature abused-oyster homogenate.

AS-EOH

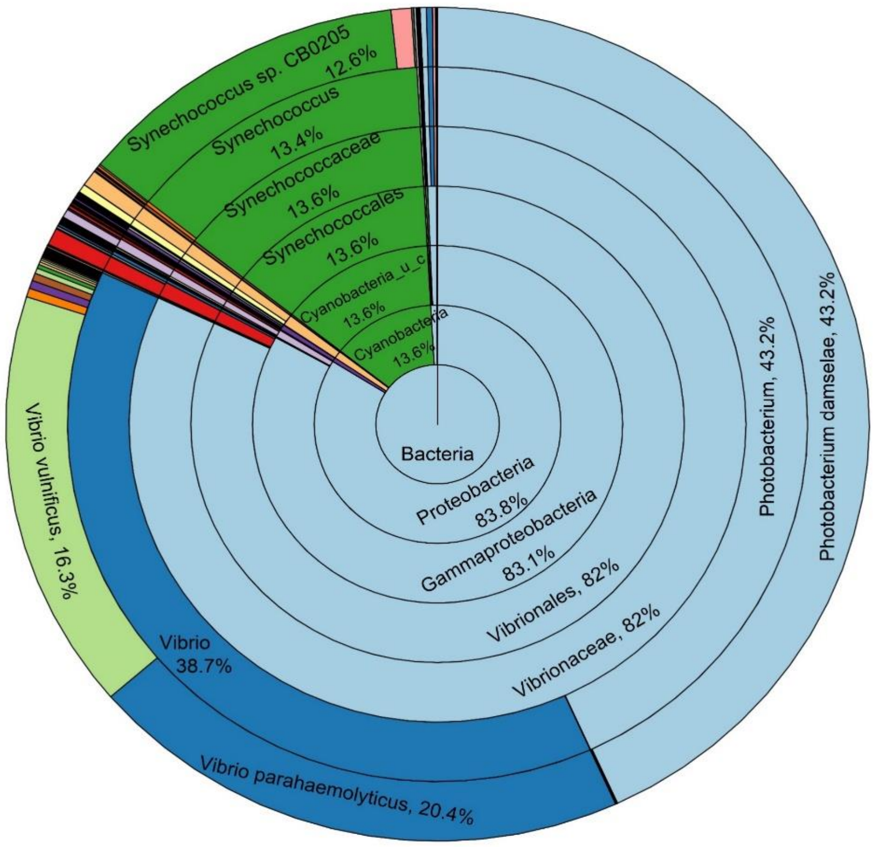

Figure S5**:** Abundance distribution of bacterial taxa in the EOH samples. AS: abundance score. EOH: Enriched-oyster homogenate.

AS-DSH

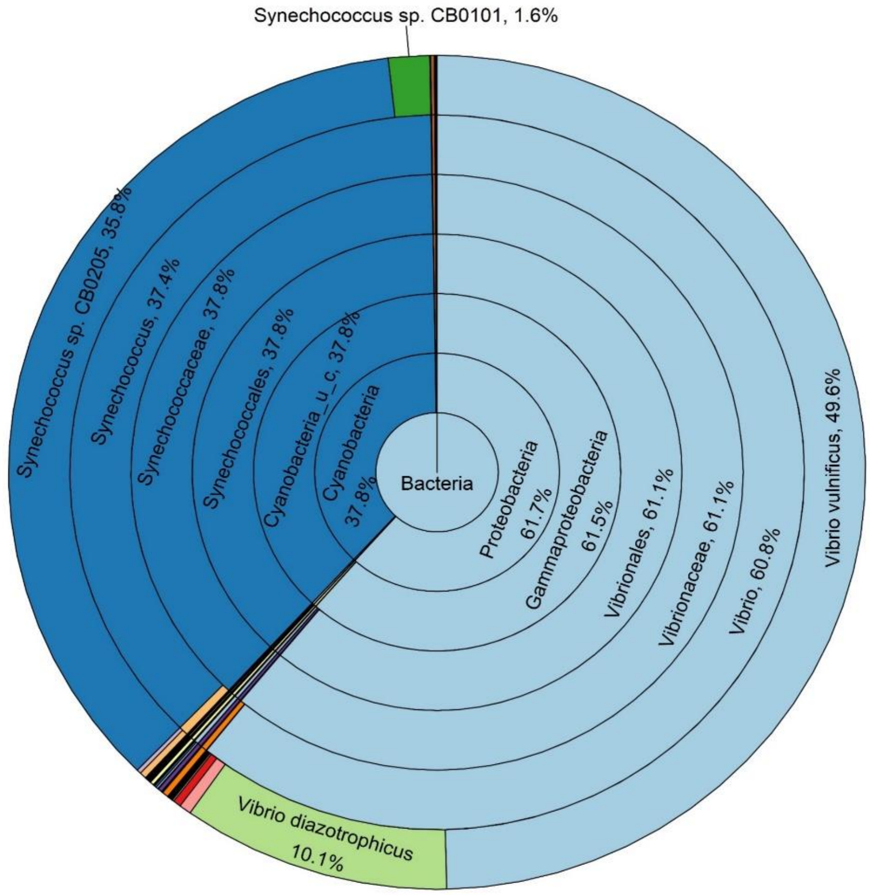

Figure S6**:** Abundance distribution of bacterial taxa in the DSH samples. AS: abundance score. DSH: Dissected stomach homogenate.

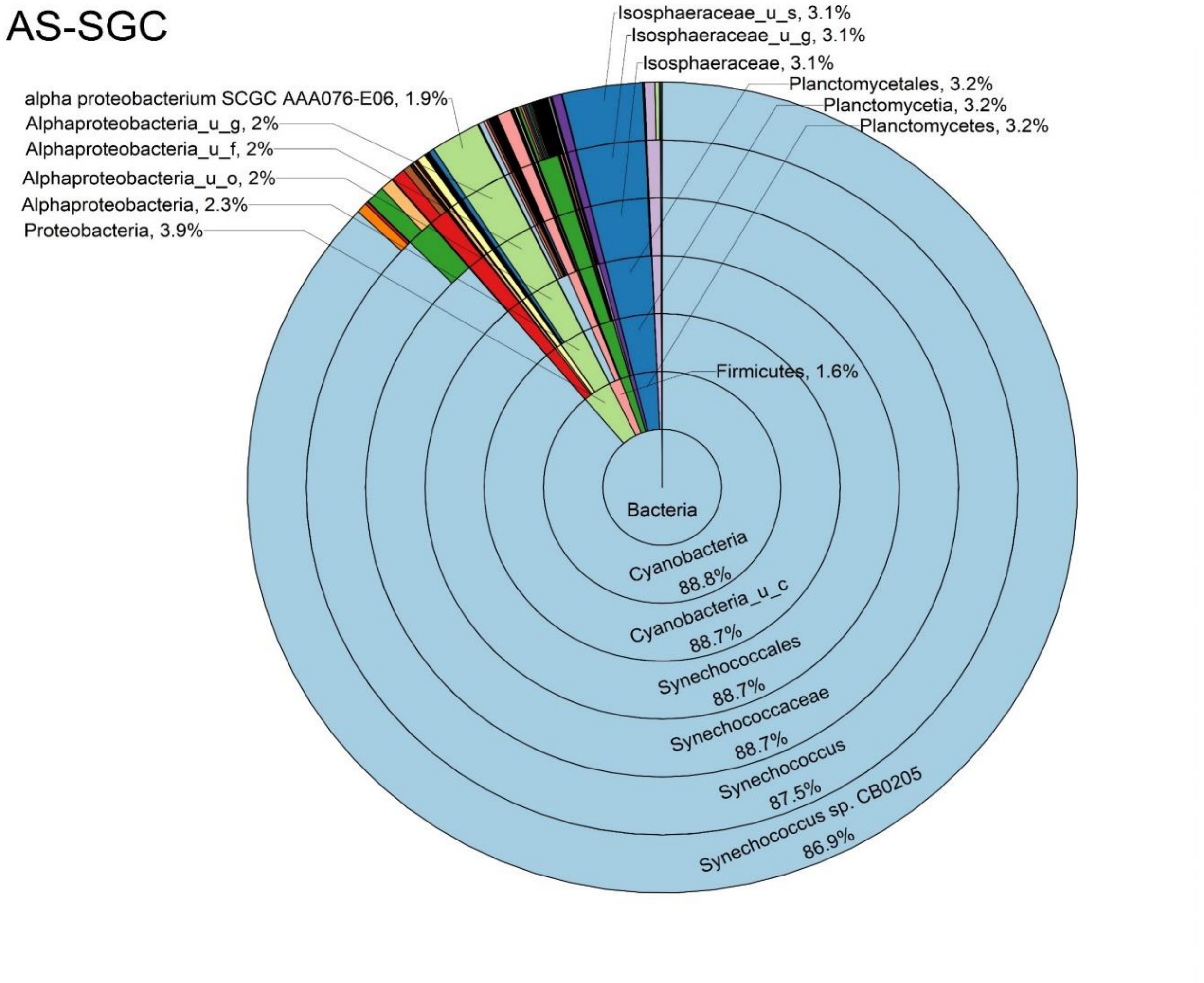

Figure S7**:** Abundance distribution of bacterial taxa in the SGC samples. AS: abundance score. SGC: Stomach gut contents.

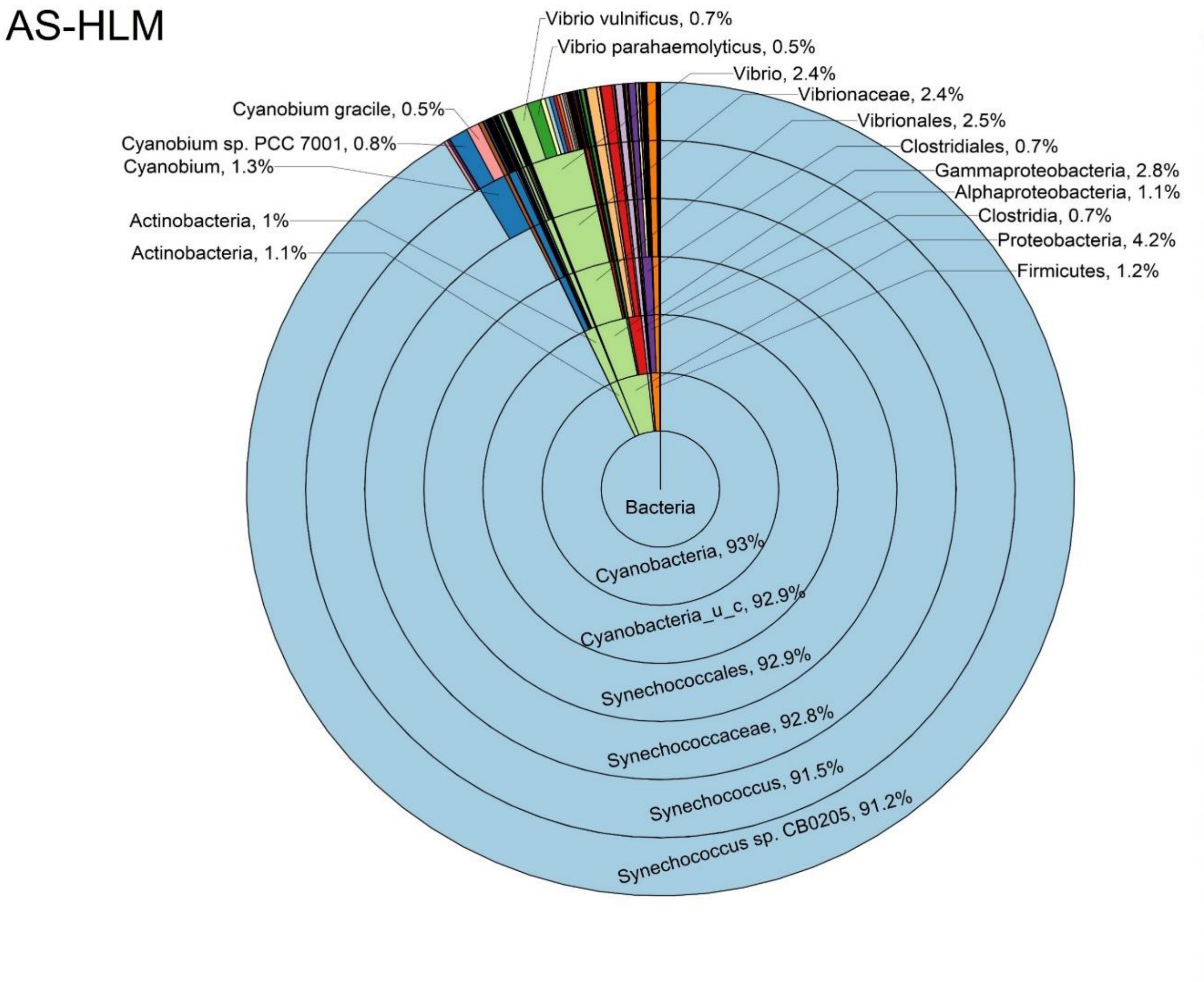

Figure S8**:** Abundance distribution of bacterial taxa in the HLM samples. AS: abundance score. HLM: Oyster-hemolymph.

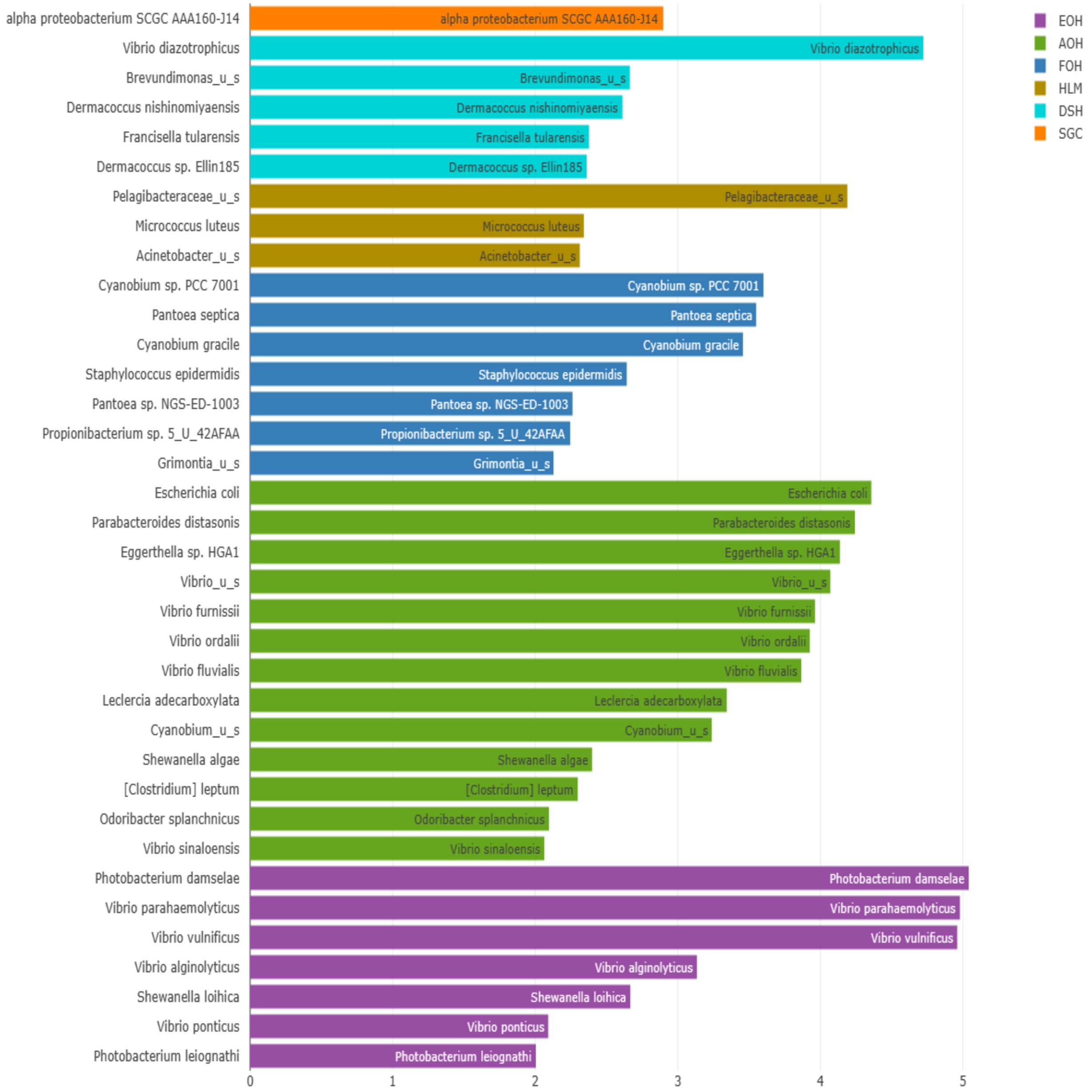

Figure S9**:** Distinctive bacterial taxa in relation to oyster sample types.

FOH: Fresh-oyster homogenate; AOH: Temperature abused-oyster homogenate; EOH: Enriched-oyster homogenate; DSH: Dissected stomach homogenate; SGC: Stomach gut contents; HLM: Oyster-hemolymph.

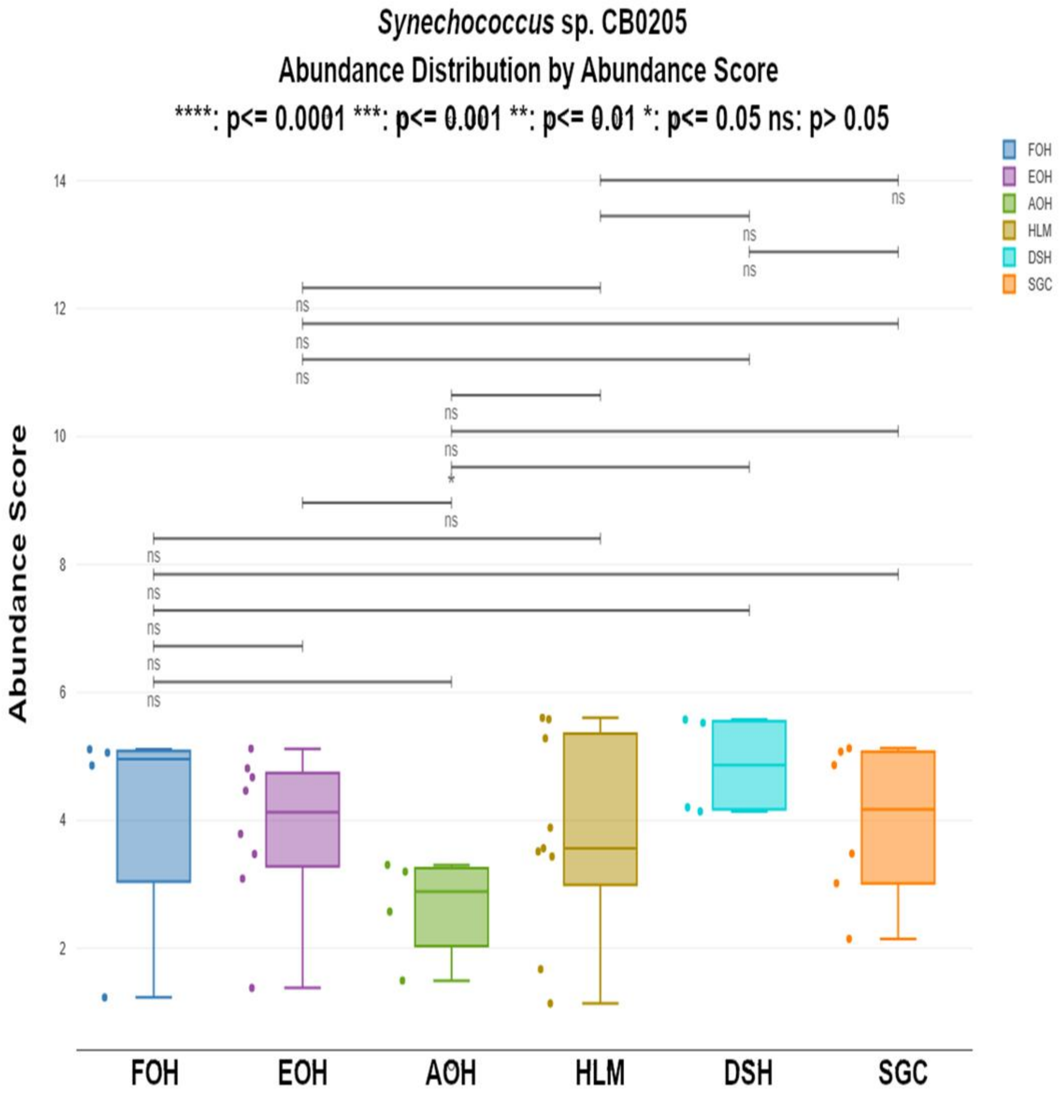

Figure S10**:** Log abundance distribution of *Synechococcus* sp. CB0205 across all sample types.

FOH: Fresh-oyster homogenate; AOH: Temperature abused-oyster homogenate; EOH: Enriched-oyster homogenate; DSH: Dissected stomach homogenate; SGC: Stomach gut contents; HLM: Oyster-hemolymph.

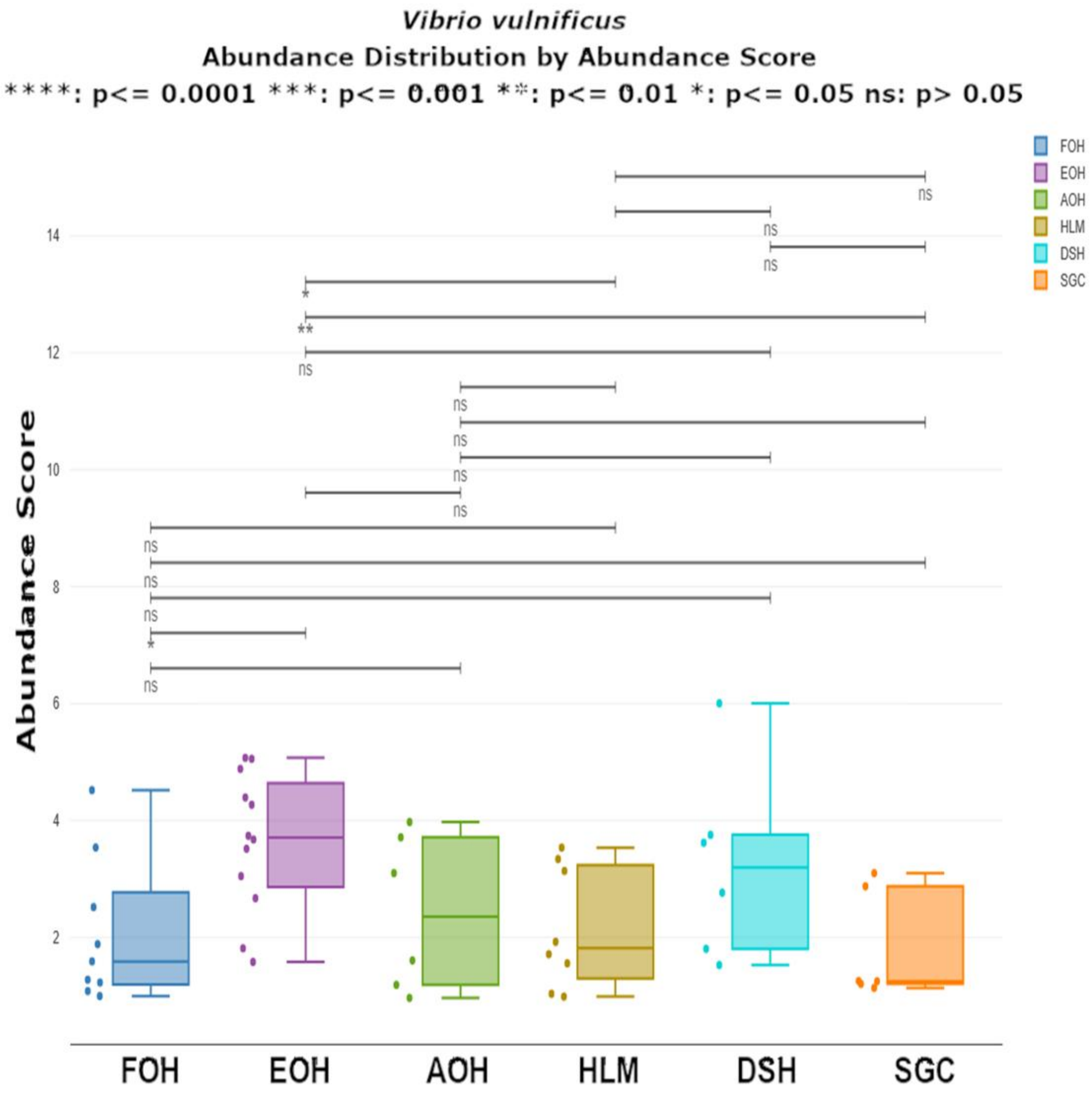

Figure S11**:** Log abundance distribution of *Vibrio vulnificus* across all sample types.

FOH: Fresh-oyster homogenate; AOH: Temperature abused-oyster homogenate; EOH: Enriched-oyster homogenate; DSH: Dissected stomach homogenate; SGC: Stomach gut contents; HLM: Oyster-hemolymph.

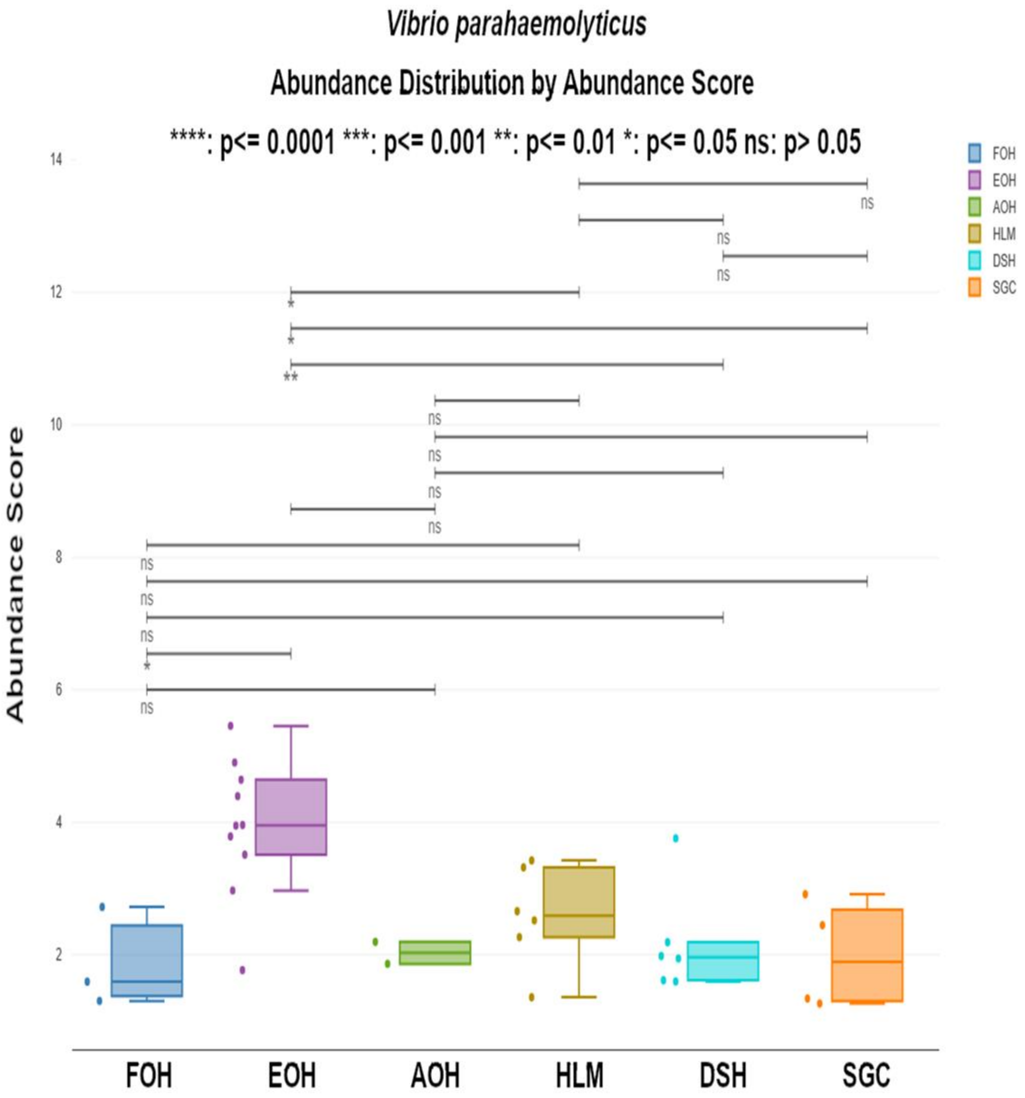

Figure S12**:** Log abundance distribution of *Vibrio parahaemolyticus* across all sample types.

FOH: Fresh-oyster homogenate; AOH: Temperature abused-oyster homogenate; EOH: Enriched-oyster homogenate; DSH: Dissected stomach homogenate; SGC: Stomach gut contents; HLM: Oyster-hemolymph.

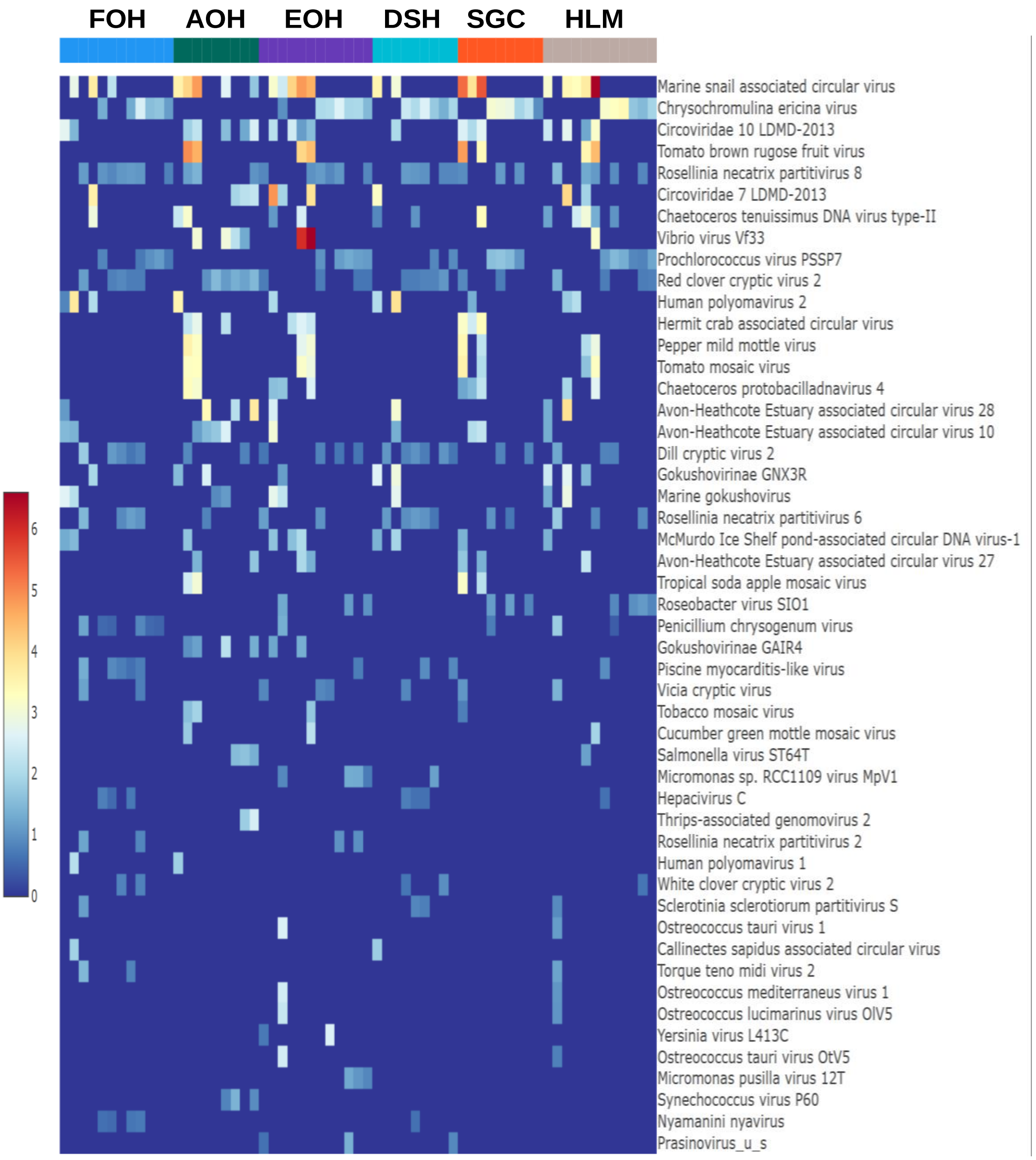

Figure S13: Mean log-abundance of viruses across all sample types.

FOH: Fresh-oyster homogenate; AOH: Temperature abused-oyster homogenate; EOH: Enriched-oyster homogenate; DSH: Dissected stomach homogenate; SGC: Stomach gut contents; HLM: Oyster-hemolymph.

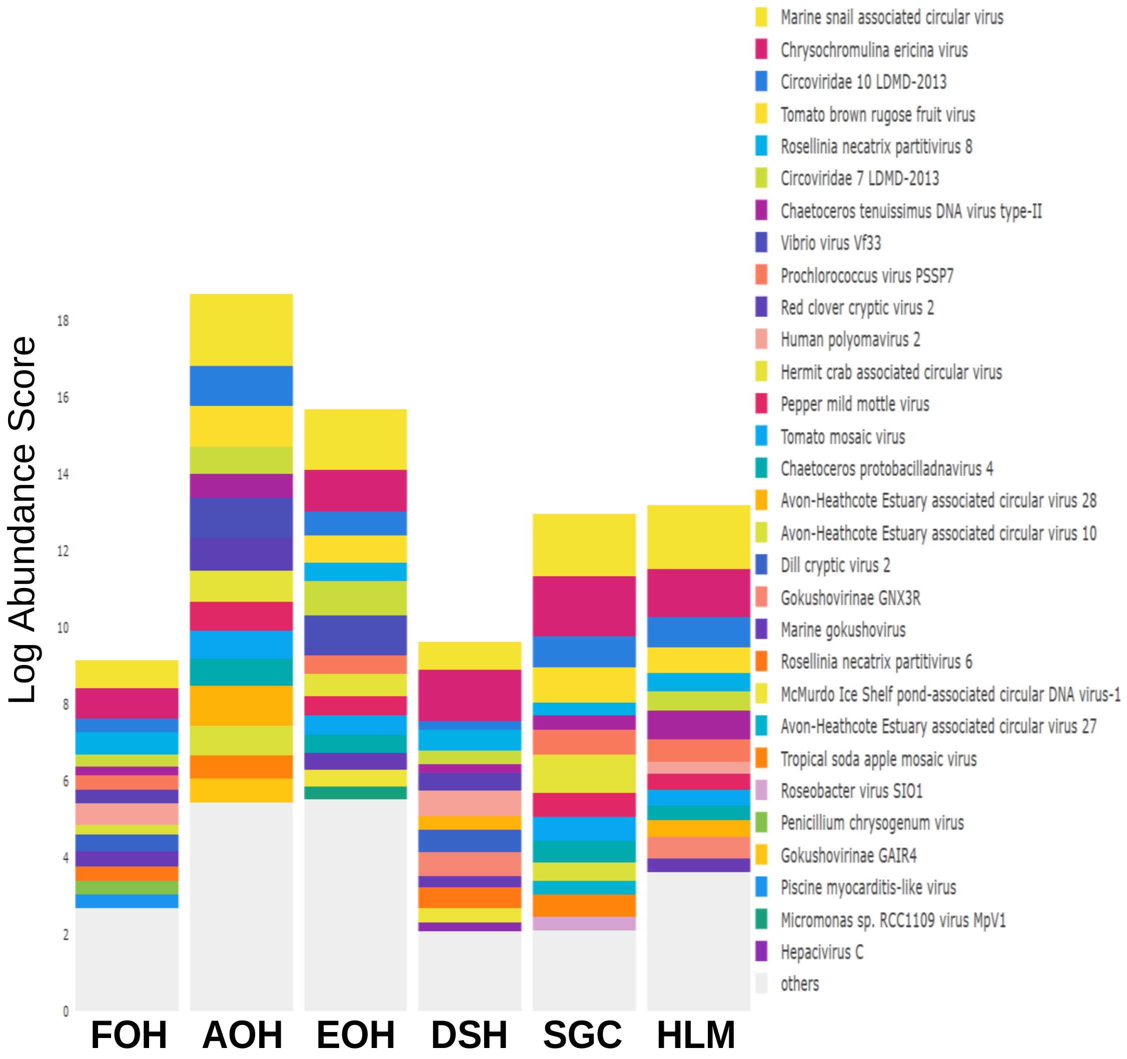

Figure S14: Relative log-abundance of viruses per sample type.

FOH: Fresh-oyster homogenate; AOH: Temperature abused-oyster homogenate; EOH: Enriched-oyster homogenate; DSH: Dissected stomach homogenate; SGC: Stomach gut contents; HLM: Oyster-hemolymph.

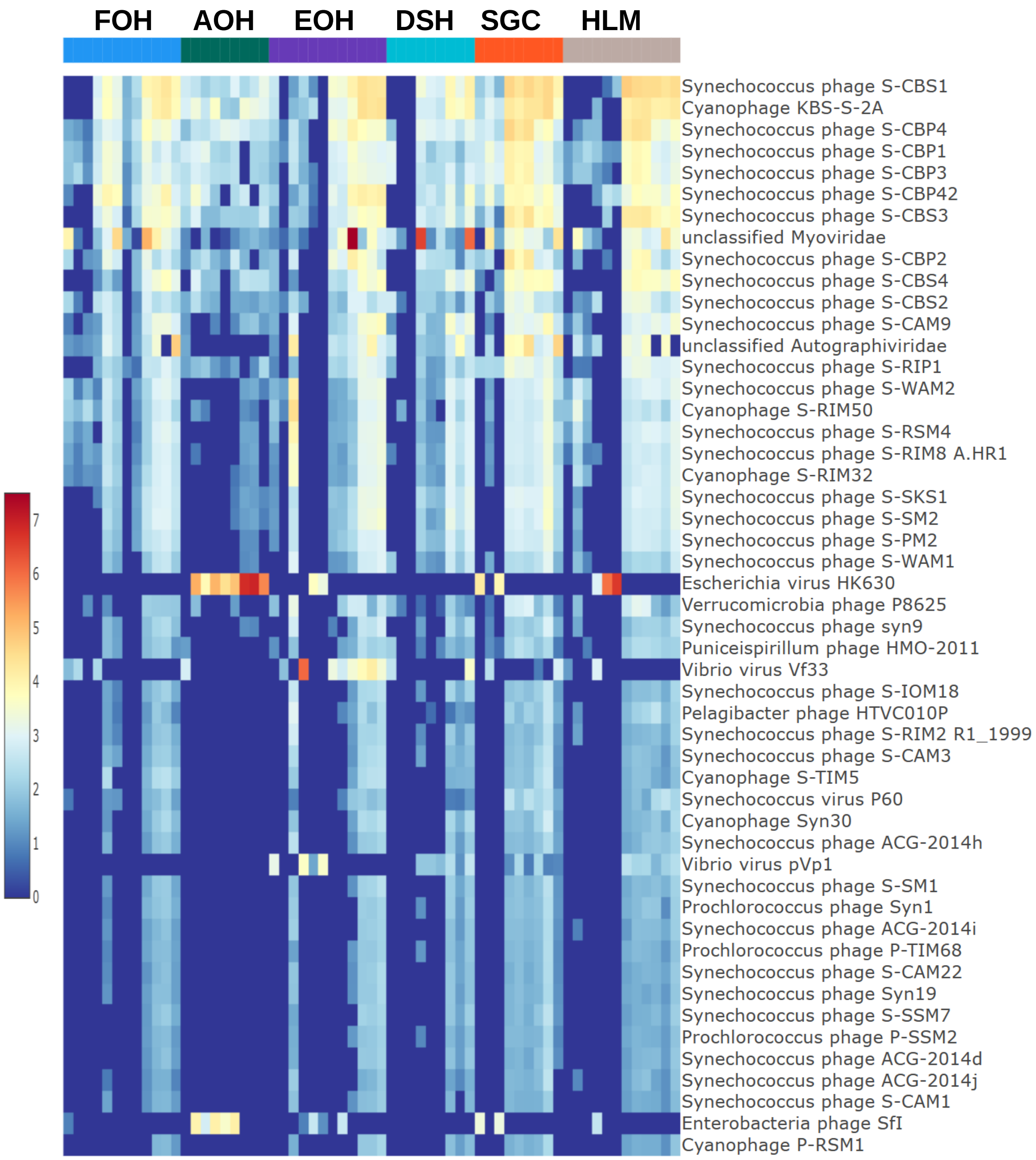

Figure S15: Mean log-abundance of phages across all sample types.

FOH: Fresh-oyster homogenate; AOH: Temperature abused-oyster homogenate; EOH: Enriched-oyster homogenate; DSH: Dissected stomach homogenate; SGC: Stomach gut contents; HLM: Oyster-hemolymph.

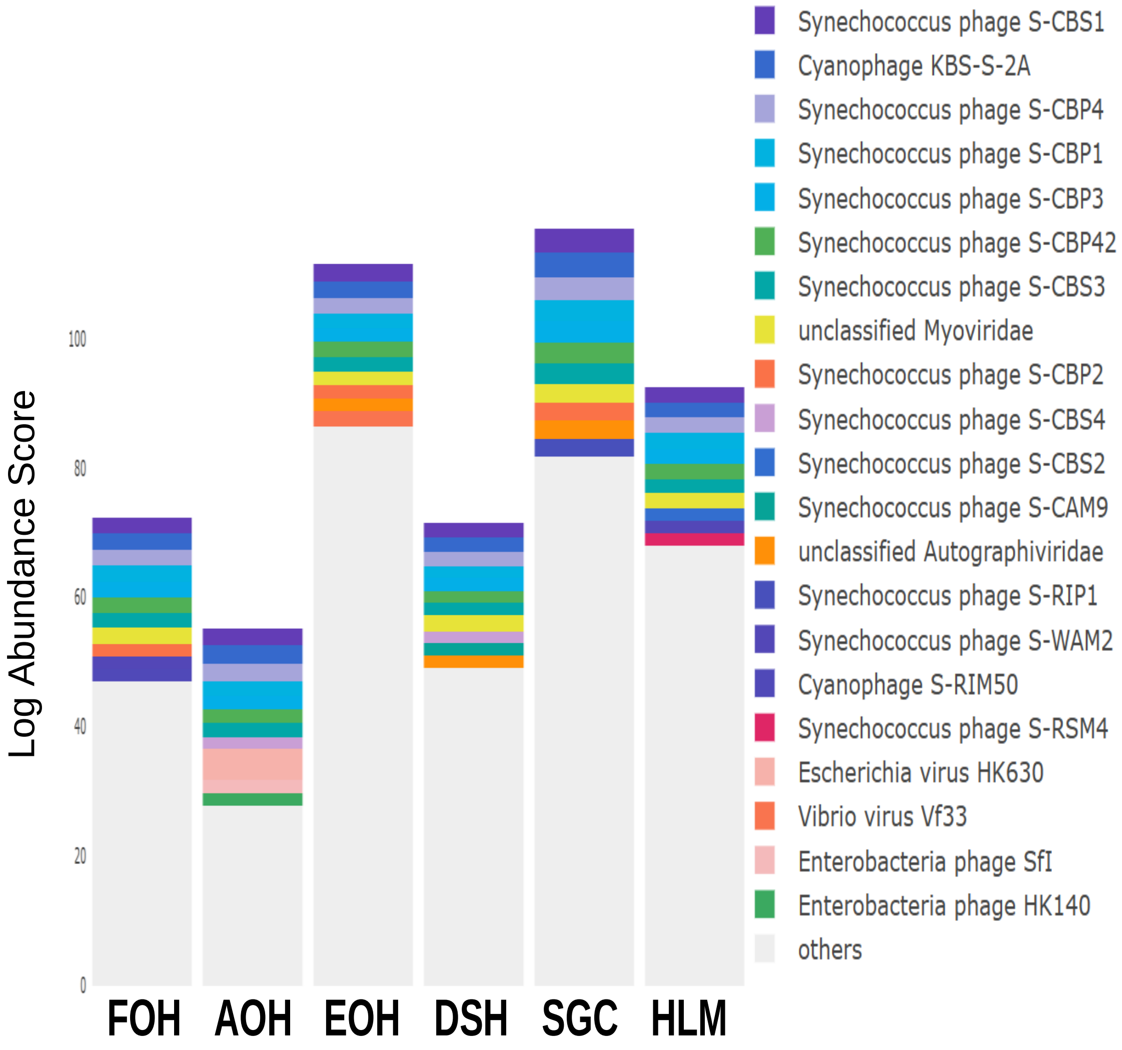

Figure S16: Relative log-abundance of phages per sample type.

FOH: Fresh-oyster homogenate; AOH: Temperature abused-oyster homogenate; EOH: Enriched-oyster homogenate; DSH: Dissected stomach homogenate; SGC: Stomach gut contents; HLM: Oyster-hemolymph.

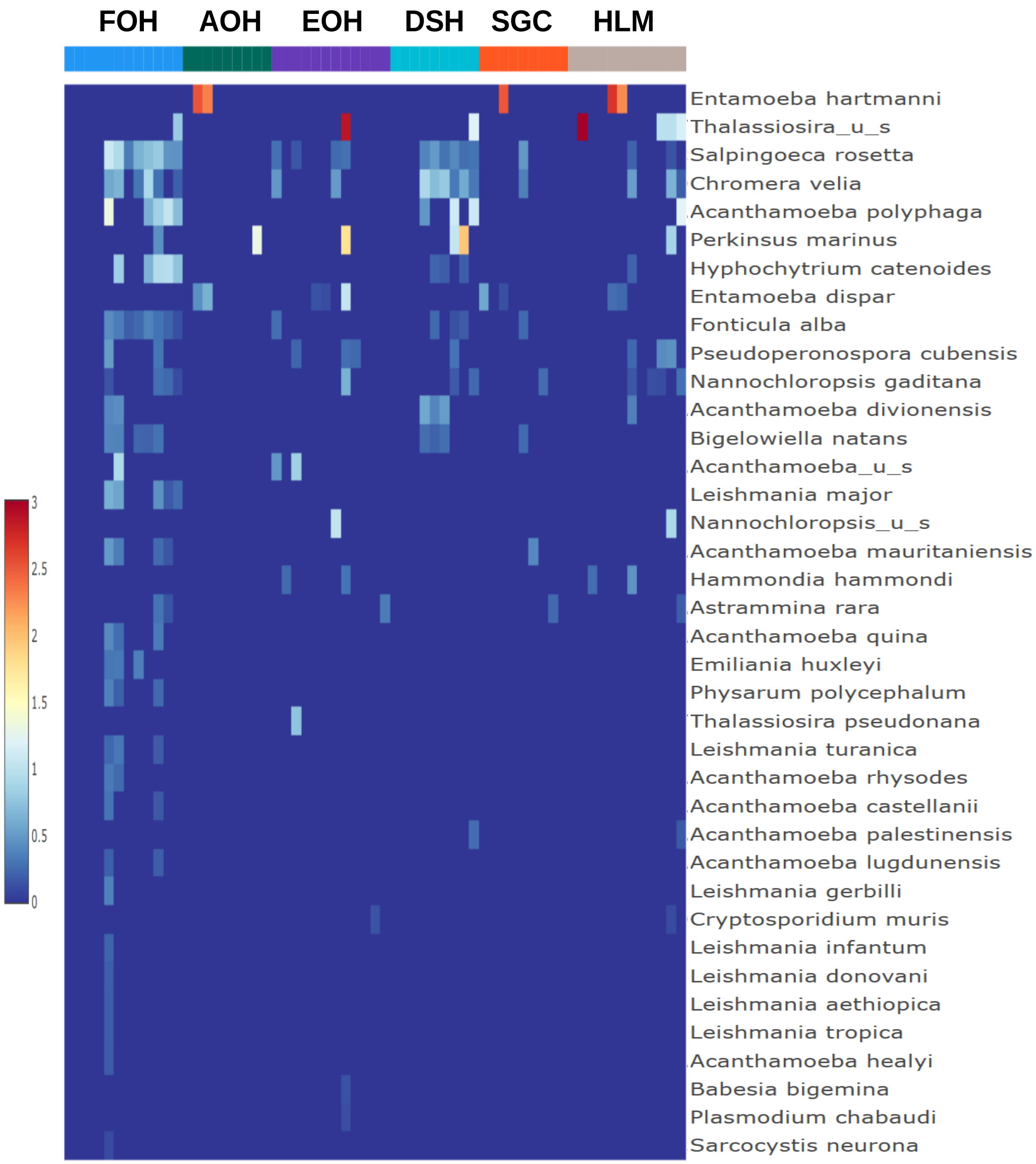

Figure S17: Mean log-abundance of protists across all sample types.

FOH: Fresh-oyster homogenate; AOH: Temperature abused-oyster homogenate; EOH: Enriched-oyster homogenate; DSH: Dissected stomach homogenate; SGC: Stomach gut contents; HLM: Oyster-hemolymph.

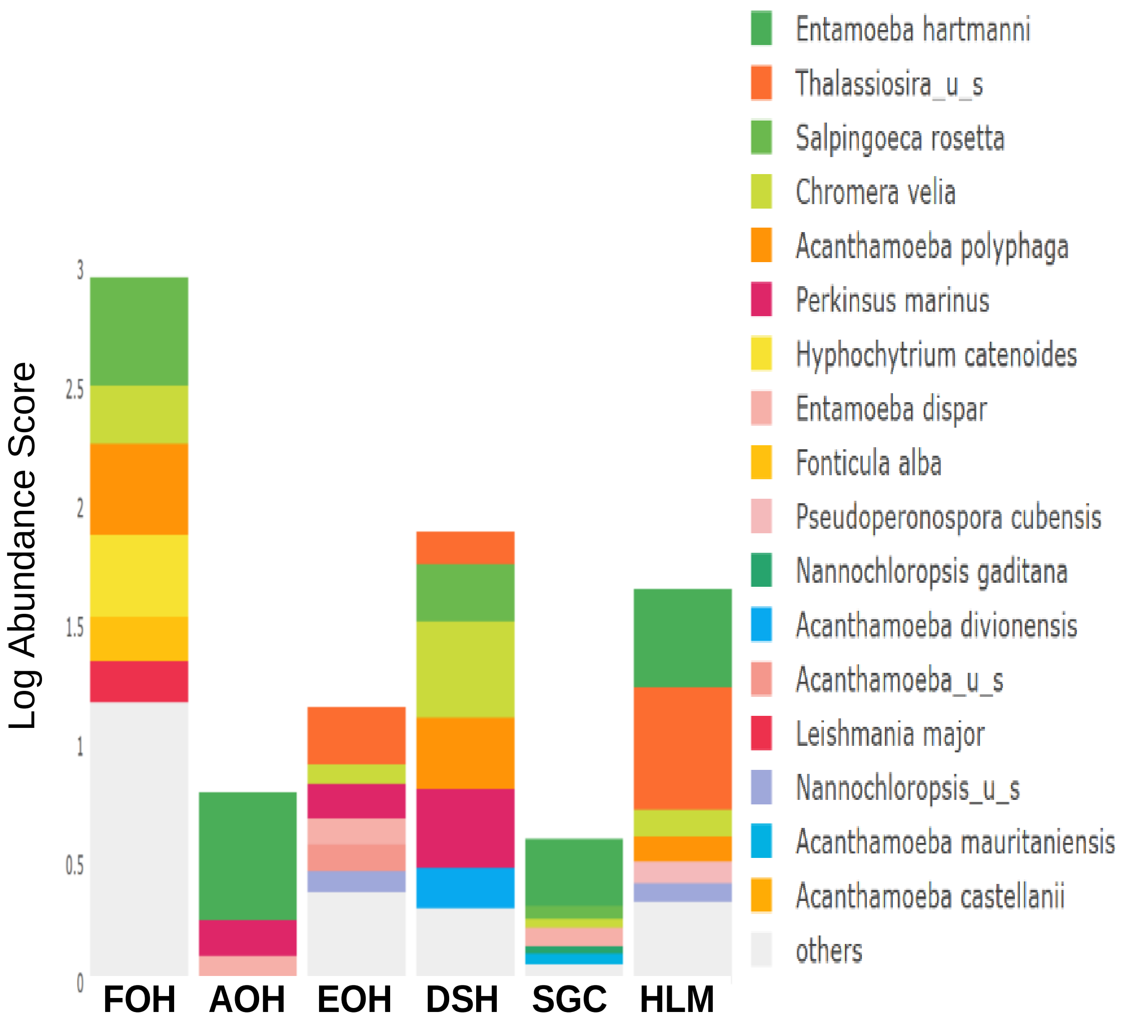

Figure S18: Relative log-abundance of protists per sample type.

FOH: Fresh-oyster homogenate; AOH: Temperature abused-oyster homogenate; EOH: Enriched-oyster homogenate; DSH: Dissected stomach homogenate; SGC: Stomach gut contents; HLM: Oyster-hemolymph.

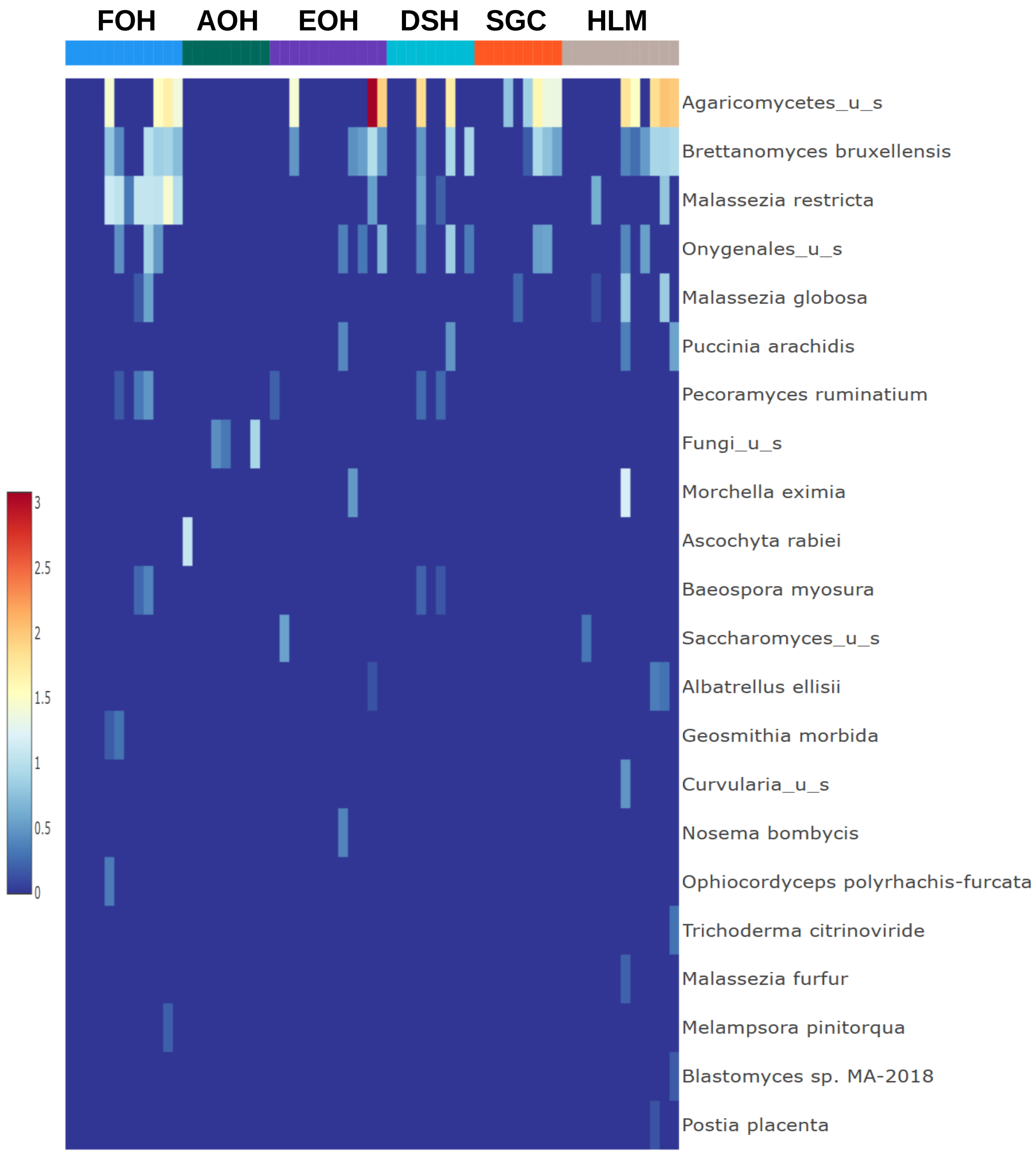

Figure S19: Mean log-abundance of fungi across all sample types.

FOH: Fresh-oyster homogenate; AOH: Temperature abused-oyster homogenate; EOH: Enriched-oyster homogenate; DSH: Dissected stomach homogenate; SGC: Stomach gut contents; HLM: Oyster-hemolymph.

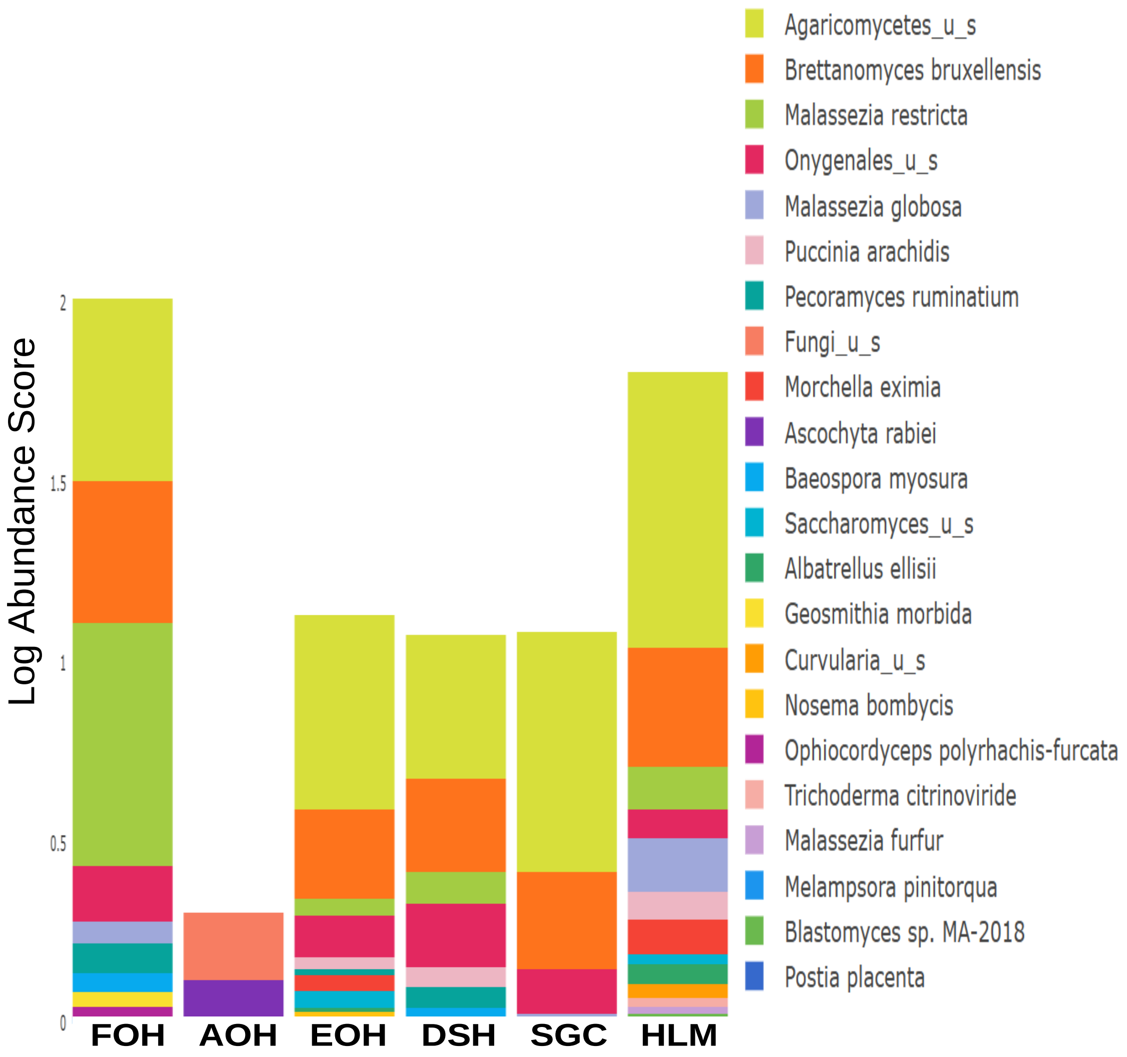

Figure S20: Relative log-abundance of fungi per sample type.

FOH: Fresh-oyster homogenate; AOH: Temperature abused-oyster homogenate; EOH: Enriched-oyster homogenate; DSH: Dissected stomach homogenate; SGC: Stomach gut contents; HLM: Oyster-hemolymph.

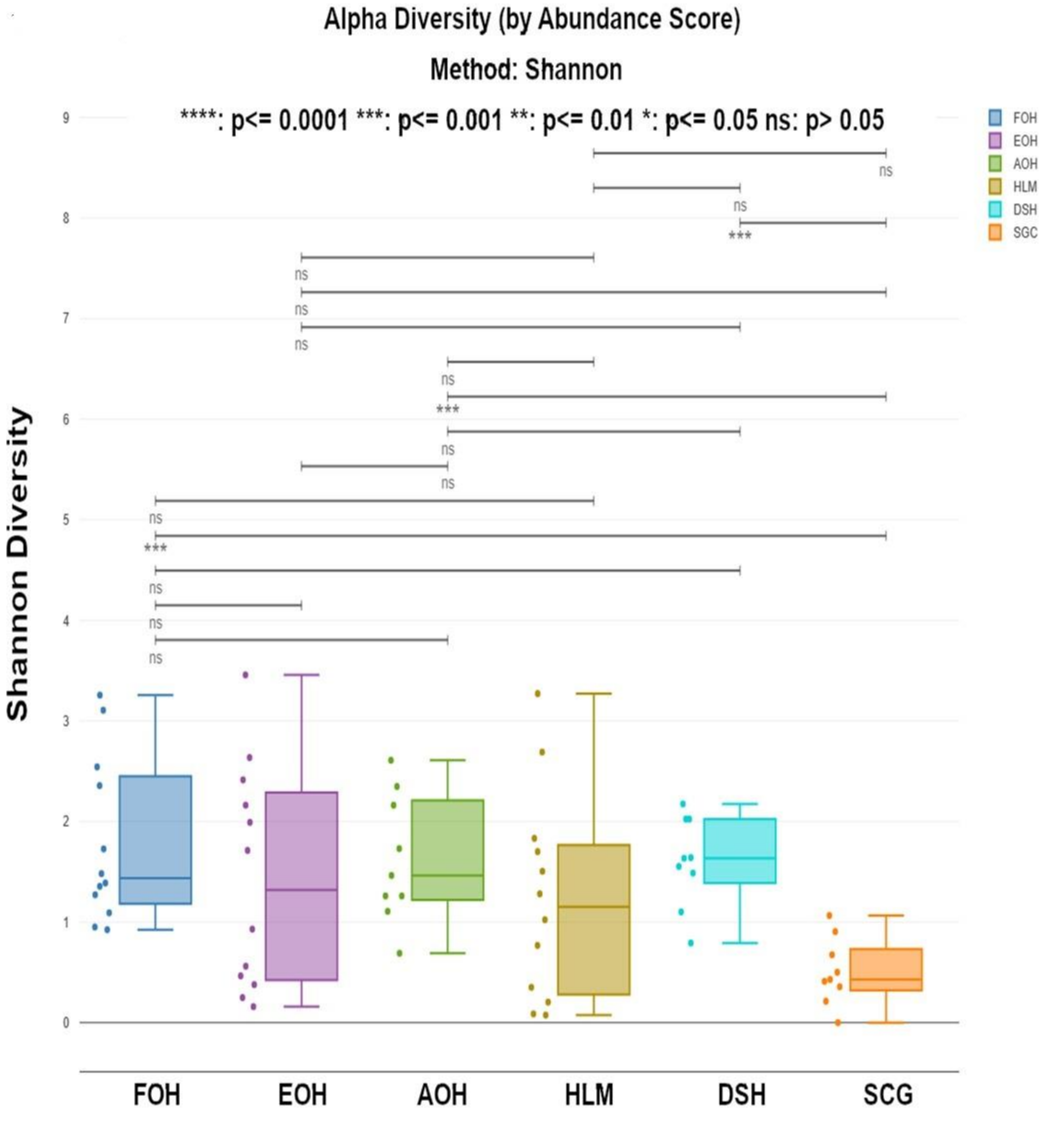

Figure S21**:** Shannon index representing the richness and evenness of viruses within all sample types. Each dot represents the Shannon diversity value for an individual sample. Statistical comparisons of medians across sample types were performed using a Wilcoxon rank-sum test.

FOH: Fresh-oyster homogenate; AOH: Temperature abused-oyster homogenate; EOH: Enriched-oyster homogenate; DSH: Dissected stomach homogenate; SGC: Stomach gut contents; HLM: Oyster-hemolymph.

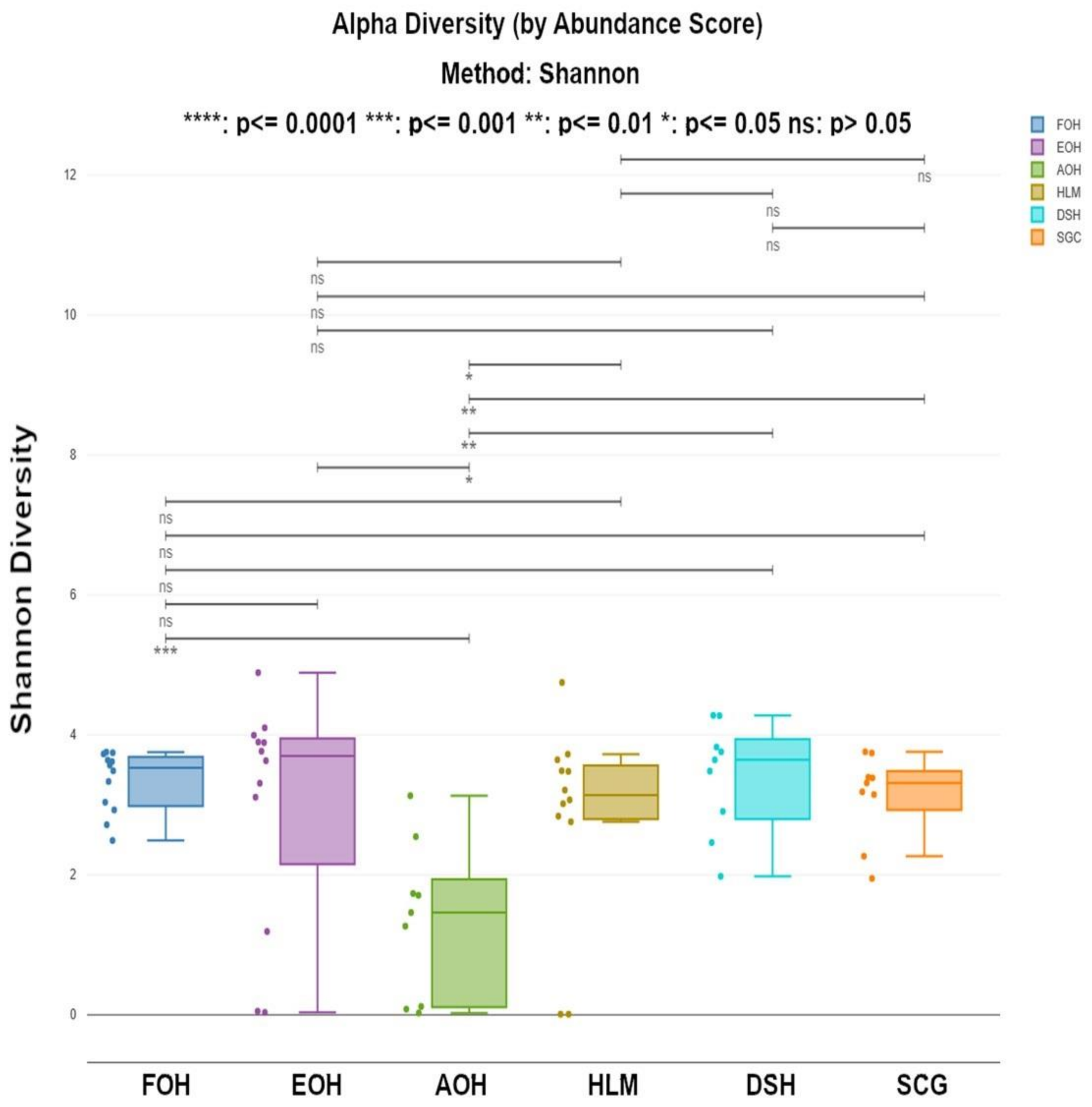

Figure S22**:** Shannon index representing the richness and evenness of phages within all sample types. Each dot represents the Shannon diversity value for an individual sample. Statistical comparisons of medians across sample types were performed using a Wilcoxon rank-sum test.

FOH: Fresh-oyster homogenate; AOH: Temperature abused-oyster homogenate; EOH: Enriched-oyster homogenate; DSH: Dissected stomach homogenate; SGC: Stomach gut contents; HLM: Oyster-hemolymph.

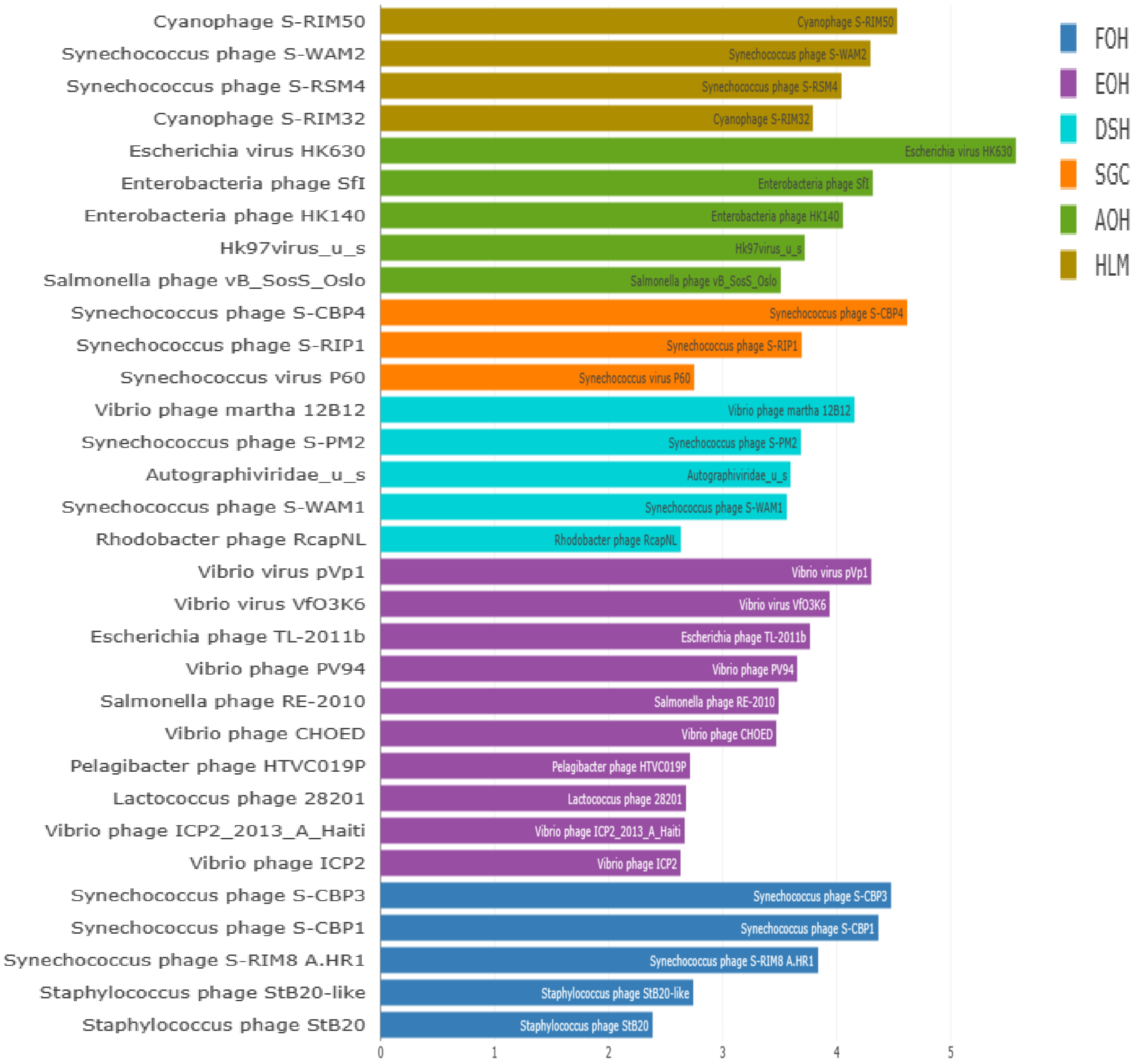

Figure S23**:** Distinctive phages in relation to oyster sample types.

FOH: Fresh-oyster homogenate; AOH: Temperature abused-oyster homogenate; EOH: Enriched-oyster homogenate; DSH: Dissected stomach homogenate; SGC: Stomach gut contents; HLM: Oyster-hemolymph.

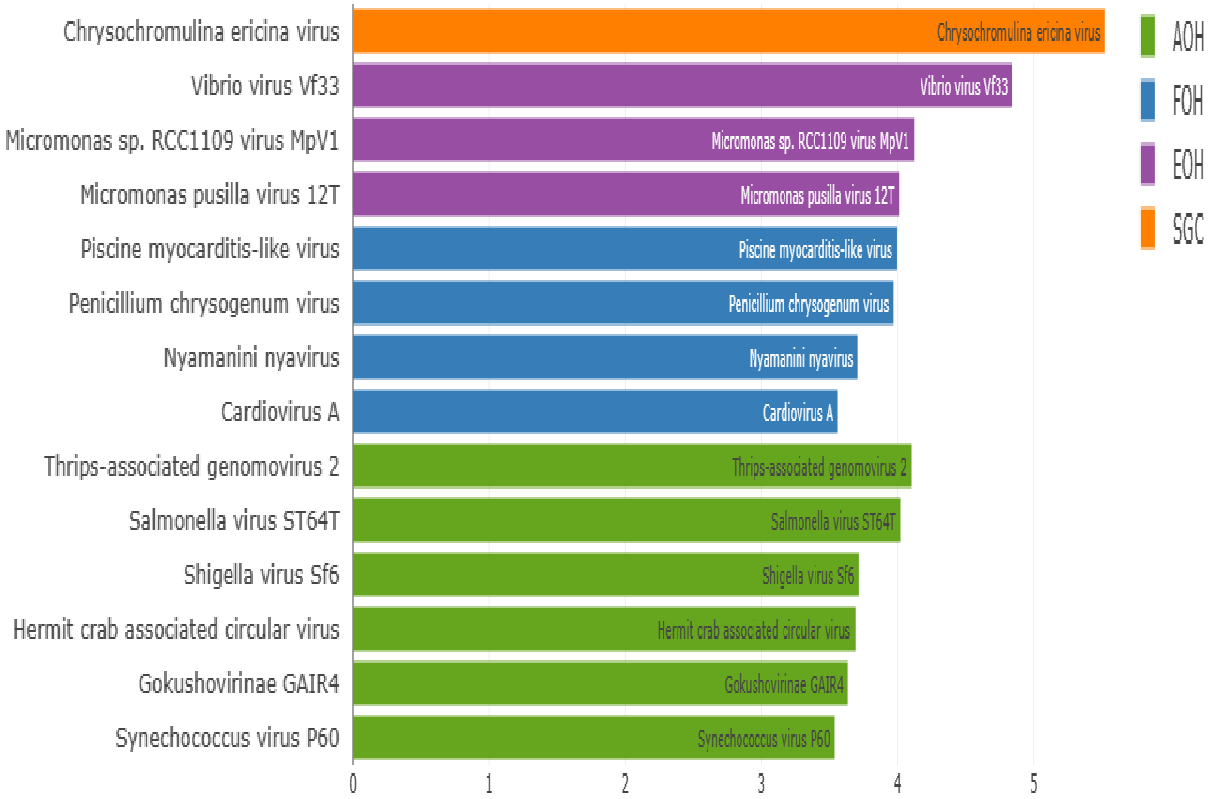

Figure S24**:** Distinctive viruses in relation to oyster sample types.

FOH: Fresh-oyster homogenate; AOH: Temperature abused-oyster homogenate; EOH: Enriched-oyster homogenate; SGC: Stomach gut contents.

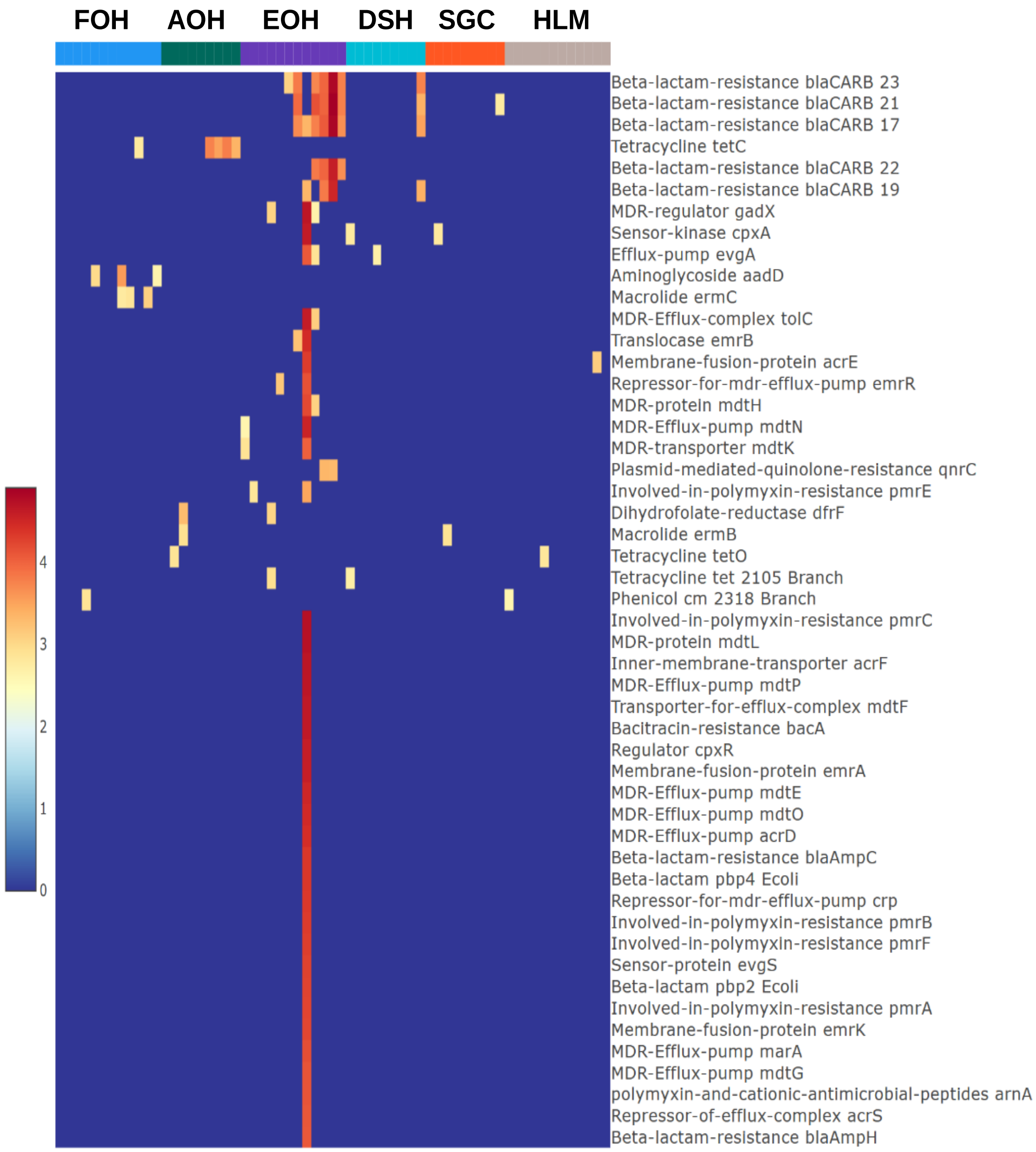

Figure S25: Mean log-abundance of antimicrobial resistance genes across all sample types.

FOH: Fresh-oyster homogenate; AOH: Temperature abused-oyster homogenate; EOH: Enriched-oyster homogenate; DSH: Dissected stomach homogenate; SGC: Stomach gut contents; HLM: Oyster-hemolymph.

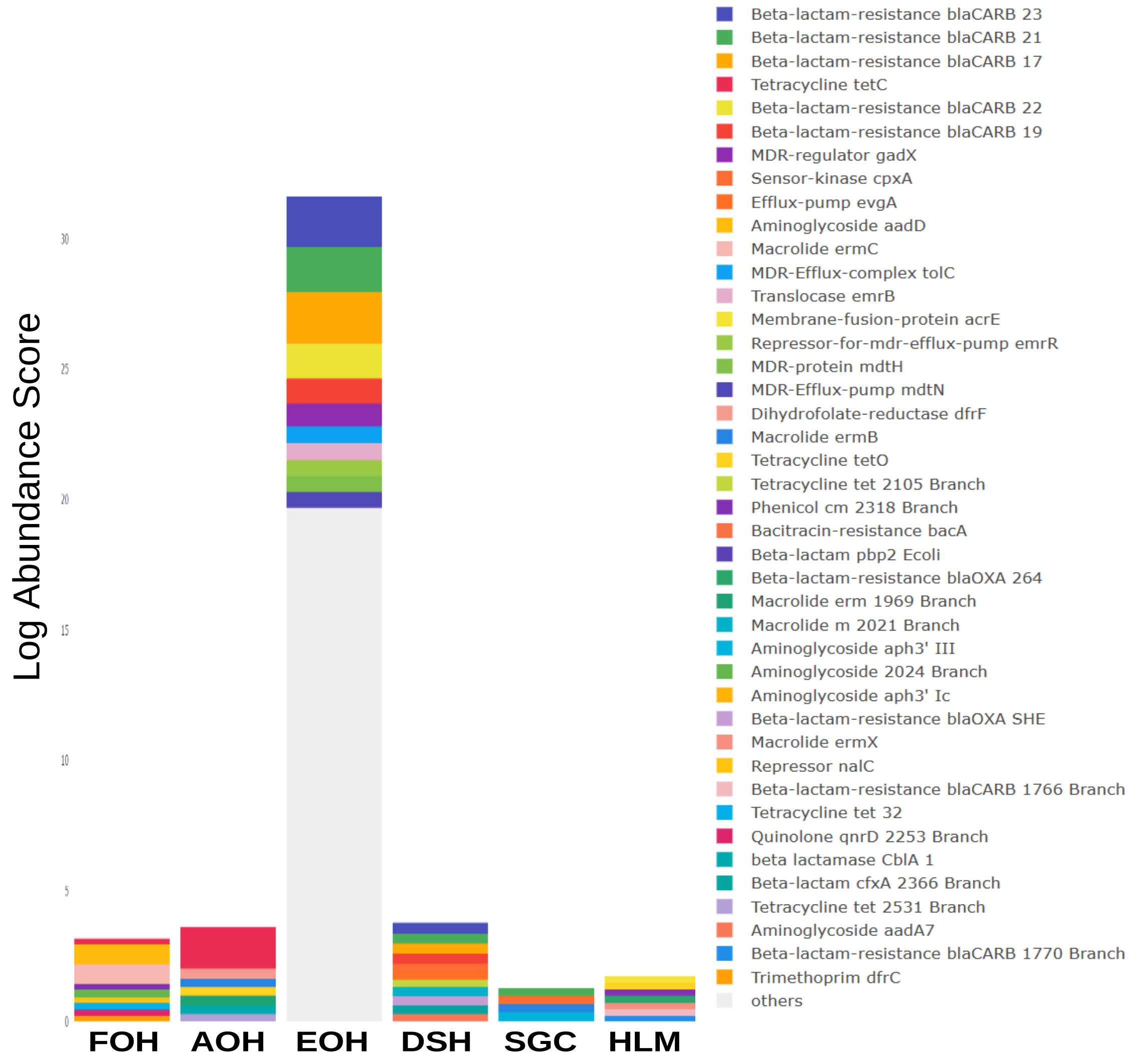

Figure S26: Relative log-abundance of antimicrobial resistance genes per sample type.

FOH: Fresh-oyster homogenate; AOH: Temperature abused-oyster homogenate; EOH: Enriched-oyster homogenate; DSH: Dissected stomach homogenate; SGC: Stomach gut contents; HLM: Oyster-hemolymph.

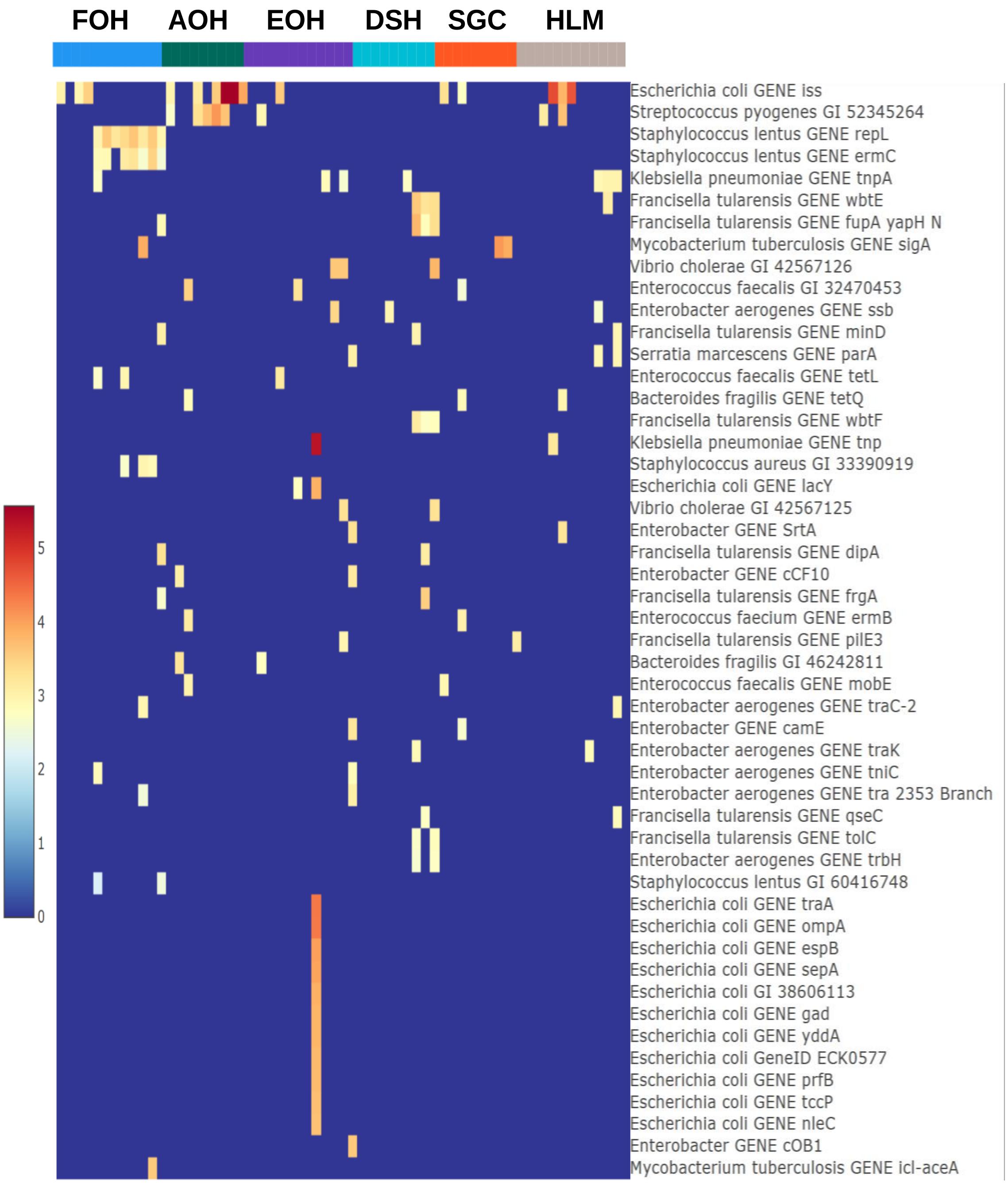

Figure S27: Mean log-abundance of virulence factor genes across all sample types.

FOH: Fresh-oyster homogenate; AOH: Temperature abused-oyster homogenate; EOH: Enriched-oyster homogenate; DSH: Dissected stomach homogenate; SGC: Stomach gut contents; HLM: Oyster-hemolymph.

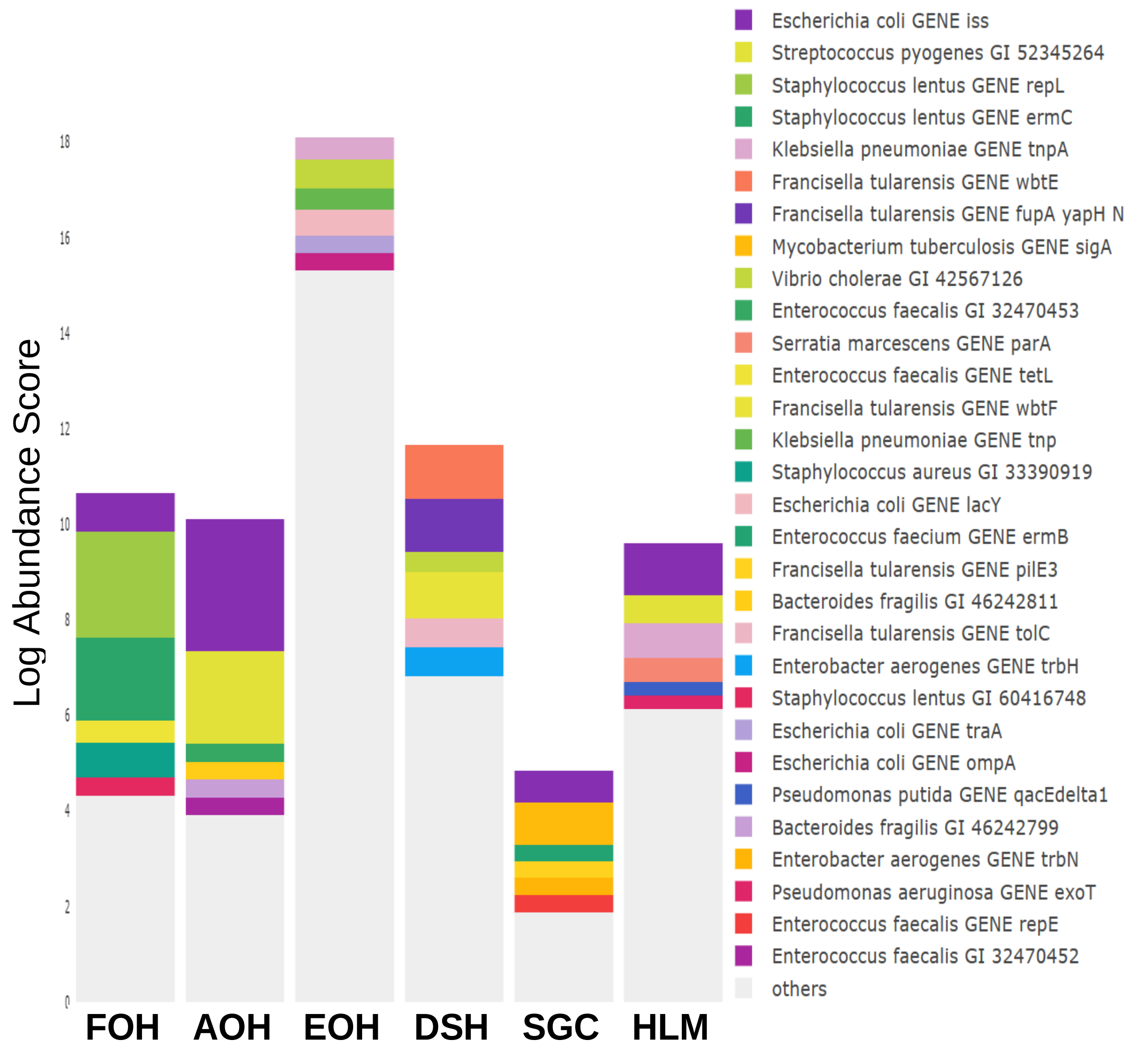

Figure S28: Relative log-abundance of virulence factors genes per sample type.

FOH: Fresh-oyster homogenate; AOH: Temperature abused-oyster homogenate; EOH: Enriched-oyster homogenate; DSH: Dissected stomach homogenate; SGC: Stomach gut contents; HLM: Oyster-hemolymph.
